# Dynamin2 controls Rap1 activation and integrin clustering in human T lymphocyte adhesion

**DOI:** 10.1371/journal.pone.0172443

**Published:** 2017-03-08

**Authors:** Felix J. Eppler, Thomas Quast, Waldemar Kolanus

**Affiliations:** Division of Molecular Immunology and Cell Biology, Life and Medical Sciences Institute (LIMES), University of Bonn, Bonn, Germany; University of Bergen, NORWAY

## Abstract

Leukocyte trafficking is crucial to facilitate efficient immune responses. Here, we report that the large GTPase dynamin2, which is generally considered to have a key role in endocytosis and membrane remodeling, is an essential regulator of integrin-dependent human T lymphocyte adhesion and migration. Chemical inhibition or knockdown of dynamin2 expression significantly reduced integrin-dependent T cell adhesion *in vitro*. This phenotype was not observed when T cells were treated with various chemical inhibitors which abrogate endocytosis or actin polymerization. We furthermore detected dynamin2 in signaling complexes and propose that it controls T cell adhesion via FAK/Pyk2- and RapGEF1-mediated Rap1 activation. In addition, the dynamin2 inhibitor-induced reduction of lymphocyte adhesion can be rescued by Rap1a overexpression. We demonstrate that the dynamin2 effect on T cell adhesion does not involve integrin affinity regulation but instead relies on its ability to modulate integrin valency. Taken together, we suggest a previously unidentified role of dynamin2 in the regulation of integrin-mediated lymphocyte adhesion via a Rap1 signaling pathway.

## Introduction

T lymphocytes are crucial for proper adaptive immune responses. Their function in homeostasis and inflammation strongly depends on their ability to adapt their adhesive state. Rapid switches of this state are achieved by regulating the main adhesion receptors of lymphocytes, the integrins [1]. As several diseases are tightly connected with leukocyte integrins, studies aiming for further details in their regulation are still of huge interest [2].

In resting T cells, leukocyte-specific integrins like lymphocyte function-associated antigen 1 (LFA-1, integrin alpha_L_/beta_2_, CD11a/CD18) or very late antigen 4 (VLA-4, integrin alpha_4_/beta_1_, CD49d/CD29) are naturally found in an inactive state. However, T lymphocytes are able to control integrin activity fast and precisely in response to their given environment and specific stimuli, a process termed “inside-out” integrin activation [3]. This may be achieved by conformational changes in integrin molecules. Extracellular integrin domains undergo transitions from bent low affinity to extended high affinity conformations, triggered by separation of the alpha- and beta-cytoplasmic integrin domains [4,5]. Several activating adapter proteins such as talin and the kindlins have been implicated in this integrin affinity regulation [6,7]. However, valency regulation also strongly influences the activation of integrins, e.g. via clustering, resulting in increased ligand binding through higher avidity and thereby enabling functional cell adhesion [8,9]. Although integrin activation has been studied extensively, the contributions and spatiotemporal patterns of affinity and valency regulation still are controversially debated [10–14].

Rap1 (Ras-related protein 1), a small GTPase of the Ras superfamily (two isoforms in mammals, Rap1a and Rap1b), is an important regulator of integrin activity and adhesion in leukocytes [15–18]. The inactive, GDP-bound form of Rap1 is activated by exchange of GDP for GTP by guanine nucleotide exchange factors (GEFs) like RapGEF1 (C3G) [19]. The activated protein interacts with several downstream effectors (e.g. RapL, RIAM or RalGDS) to control diverse cellular events [20,21].

Dynamin2, a large GTPase in the family of dynamin-like proteins, has mainly been studied for its role in membrane fission and endocytosis, where it forms oligomers to abscise newly formed endocytic pits [22]. Apart from that, dynamin2 has been described to regulate also other important cellular functions, including cytokinesis, cytoskeletal dynamics and mesenchymal cell migration [23–26]. In addition, dynamin2 has been shown to control the assembly, turnover and dynamics of podosomes, invadopodia and mature focal adhesions [27–32]. However, these complex and persistent adhesion structures are absent in amoeboid cells like lymphocytes and mainly formed in strongly adherent and slowly migrating cell types [33–35]. Dynamin2 was furthermore described as a regulator of T cell activation by controlling actin polymerization at the immunological synapse and by sustaining T cell receptor (TCR) signaling via its function in endocytosis [36,37]. Mice harboring a T cell-specific dynamin2 knockout show disordered trafficking of thymocytes and T cells resulting in lymphopenia, which was explained by defective sphingosine-1-phosphate receptor 1 signaling [38]. However, the role of dynamin2 in integrin-mediated lymphocyte adhesion and migration has not been studied so far.

Here, we investigated the function of dynamin2 in integrin activation and adhesion in human lymphocytes. Primary resting CD4^+^ T cells either treated with chemical inhibitors for dynamin2 activity or following RNAi (RNA interference) of dynamin2 showed strong defects in the activation of RapGEF1 and Rap1, leading to a loss of adhesion. Interestingly, cells lacking dynamin2 activity did not manifest a defective integrin affinity regulation. In contrast, integrin valency regulation was massively disturbed in steady state and following activation of T cells. Taken together, our data suggest an important role of dynamin2 in Rap1-dependent T cell adhesion and integrin clustering events.

## Results

### Dynamin2 regulates integrin-dependent adhesion of lymphocytes

To analyze the function of dynamin2 in lymphocyte adhesion we mainly used primary human resting CD4^+^ T cells as a model system. T cells are particularly suitable for studying leukocyte adhesion as they do not adhere to mere plastic and depend on adhesion-inducing stimuli in order to stick to specific integrin ligands (S1A and S1B Fig).

Three dynamin isoforms are described in mammalians, but we could only detect mRNA of the ubiquitously expressed dynamin2 in human T cells (S1C Fig). This allows a specific analysis of dynamin2 function in T lymphocyte adhesion and excludes compensatory effects of other dynamin isoforms. Besides, dynamin2 eGFP forms cluster-like structures at the basal plasma membrane of Jurkat E6.1 T cells adherent to a surface coated with the beta_2_-integrin ligand ICAM-1 and activating anti-CD3/CD28 antibodies, i.e. it localizes specifically to the adhesion-mediating compartment of the cell (S1D Fig; S1 Video).

**Fig 1:**
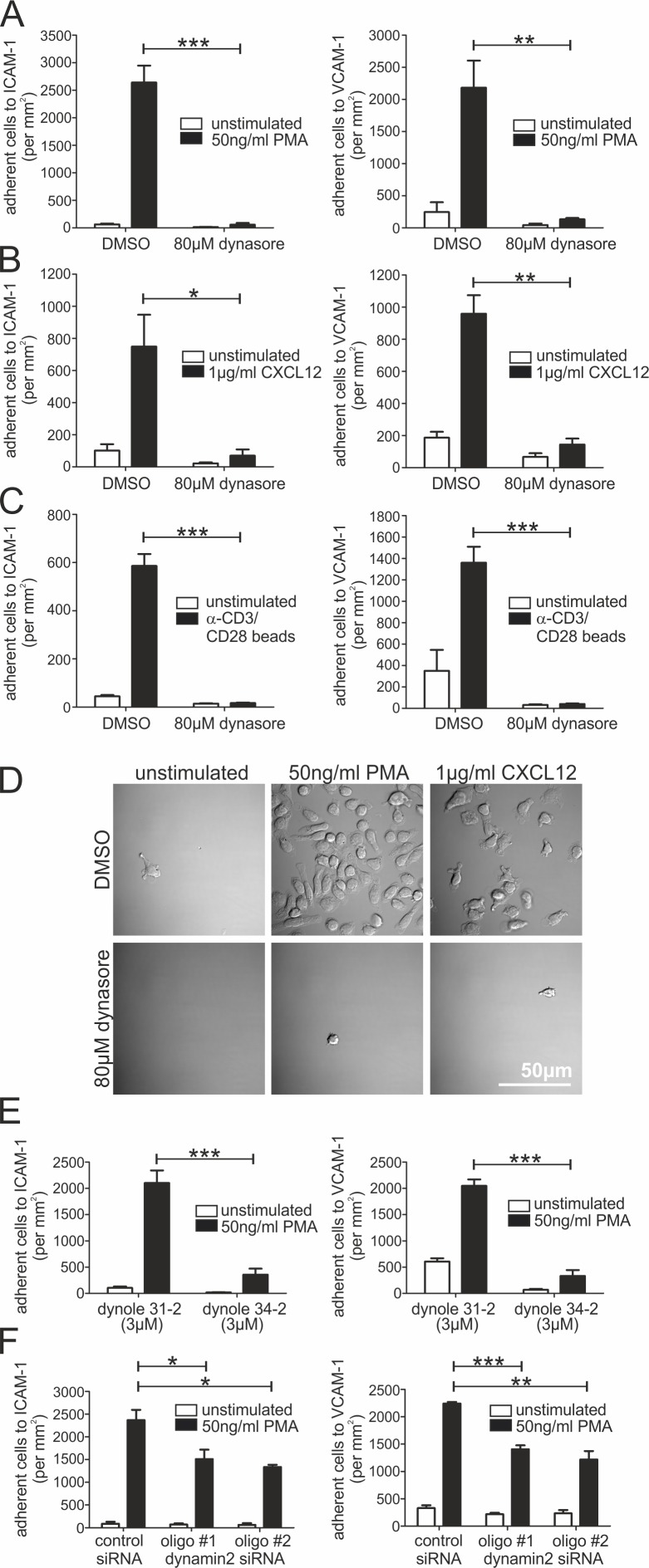
Dynamin2 is essential for integrin-mediated static adhesion of human T cells. (**A**-**C**) Analysis of the adhesion of primary human resting CD4^+^ T cells to either ICAM-1-Fc or VCAM-1-Fc under static conditions. Lymphocytes were treated with DMSO as a control or 80μM dynasore to inhibit dynamin2 activity. If indicated, adhesion was stimulated with (**A**, n = 3–5) 50ng/ml PMA, (**B**, n = 3–5) 1μg/ml CXCL12 or (**C**, n = 3) anti-CD3/CD28-coated beads (1:1 ratio to cells). 45min after seeding, the total number of adherent cells per mm^2^ was quantified. (**D**) Detailed differential interference contrast (DIC) images of representative ICAM-1-Fc coated areas with adherent CD4^+^ T lymphocytes following treatment with DMSO or dynasore and, if indicated, stimulation with PMA or CXCL12. (**E**, n = 4) Analysis of static adhesion of primary human resting CD4^+^ T cells to either ICAM-1-Fc or VCAM-1-Fc following treatment with dynole 31–2 as a control or dynole 34–2 to inhibit dynamin2 activity. If indicated, cells were stimulated with 50ng/ml PMA. 45min after seeding, the total number of adherent cells per mm^2^ was quantified. (**F**, n = 3) Analysis of static adhesion of primary human resting CD4^+^ T cells to either ICAM-1-Fc or VCAM-1-Fc 48h after the cells were transfected with either control siRNA or dynamin2 siRNA to knock down the large GTPase. If indicated, cells were stimulated with 50ng/ml PMA. 45min after seeding, the total number of adherent cells per mm^2^ was quantified. Mean +SEM, *P≤0.05, **P≤0.01, ***P≤0.001.

**Fig 2:**
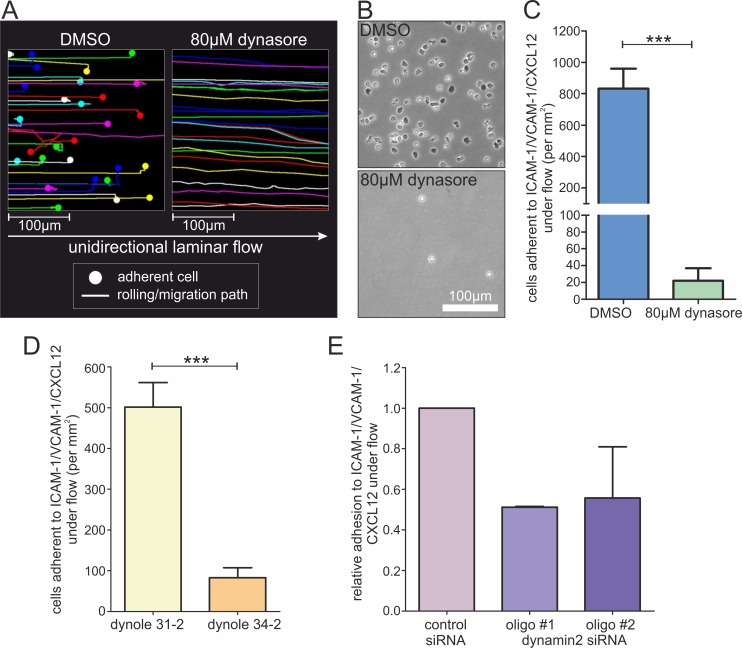
Dynamin2 regulates integrin-mediated T lymphocyte adhesion under laminar flow. Analysis of adhesion properties of primary human resting CD4^+^ T cells to a surface coated with ICAM-1-Fc and VCAM-1-Fc covered by CXCL12 under unidirectional laminar flow (**A**-**C** 2.8 dyn/cm^2^, **D**-**E** 1 dyn/cm^2^). (**A**-**C**) T Lymphocytes were either treated with DMSO as a control or with dynasore to inhibit dynamin2 activity. (**A**) Tracks of rolling and/or migrating T cells along the coated surface under unidirectional laminar flow. Points indicate adherent cells. (**B**) Phase contrast images showing representative areas of the coated surfaces with adherent T cells after 30min exposure to a cell suspension under laminar flow and subsequent washing with HBSS. (**C**) End point quantification of T lymphocyte adhesion under flow (corresponding to (**A**) and (**B**), n = 4). (**D**) End point quantification of T lymphocyte adhesion following treatment of the cells with either dynole 31–2 as a control or dynole 34–2 to inhibit dynamin2 activity (n = 6). (**E**) End point analysis of T cell adhesion after transfection of the lymphocytes with either control siRNA or siRNA oligos directed against dynamin2 (n = 2). Mean +SEM, ***P≤0.001.

To analyze whether dynamin2 activity regulates adhesion processes in lymphocytes, we assessed static adhesion of human CD4^+^ T cells to integrin ligands. Compared with DMSO-treated control cells, incubation with a small molecule inhibitor of dynamin2 GTPase activity, dynasore [39], strongly reduced PMA-, CXCL12- and anti-CD3/CD28 antibody-stimulated T cell adhesion to ICAM-1 or VCAM-1, as well as PMA-stimulated adhesion to fibronectin (Fig 1A–1D; S2A Fig). The adhesion deficiency of dynasore-treated T cells is reversible, as the cells adhered normally after washing-out the inhibitor (S2B Fig). Integrin-mediated adhesion was also dynamin2-dependent in other primary lymphocytes such as CD4^+^ effector T cells, NK cells, CD8^+^ T cells and CD19^+^ B cells (S2C–S2J Fig). To verify these findings, we used a different inhibitor of dynamin2 activity, based on an unrelated chemical scaffold [40,41]. We observed that dynole 34–2, but not dynole 31–2, a structurally related but inactive compound, significantly reduced integrin-mediated adhesion of human CD4^+^ T cells (Fig 1E). To further confirm the results, we also performed siRNA-mediated knockdown of dynamin2 in human CD4^+^ T cells. RNAi of dynamin2 significantly reduced PMA-stimulated static adhesion of CD4^+^ T cells to ICAM-1 and VCAM-1 compared to control cells (Fig 1F). Since shear stress can influence cell adhesion dynamics quite dramatically [42], we also applied microfluidics, which mimic physiological shear flow conditions of lymphocytes in blood vessels. Control cells adhered strongly to the ICAM-1/VCAM-1/CXCL12-coated microchannel surface, whereas dynasore-treated CD4^+^ T lymphocytes were not able to switch from rolling to firm adhesion (Fig 2A–2C; S2 Video). Similarly, dynole 34–2 as well as dynamin2 knockdown strongly reduced cell adhesion under flow (Fig 2D and 2E). Taken together, we conclude that dynamin2 is essential for integrin-mediated lymphocyte adhesion under both static and physiological flow conditions.

### Dynamin2 activity is essential for adhesion-dependent T cell migration

**Fig 3:**
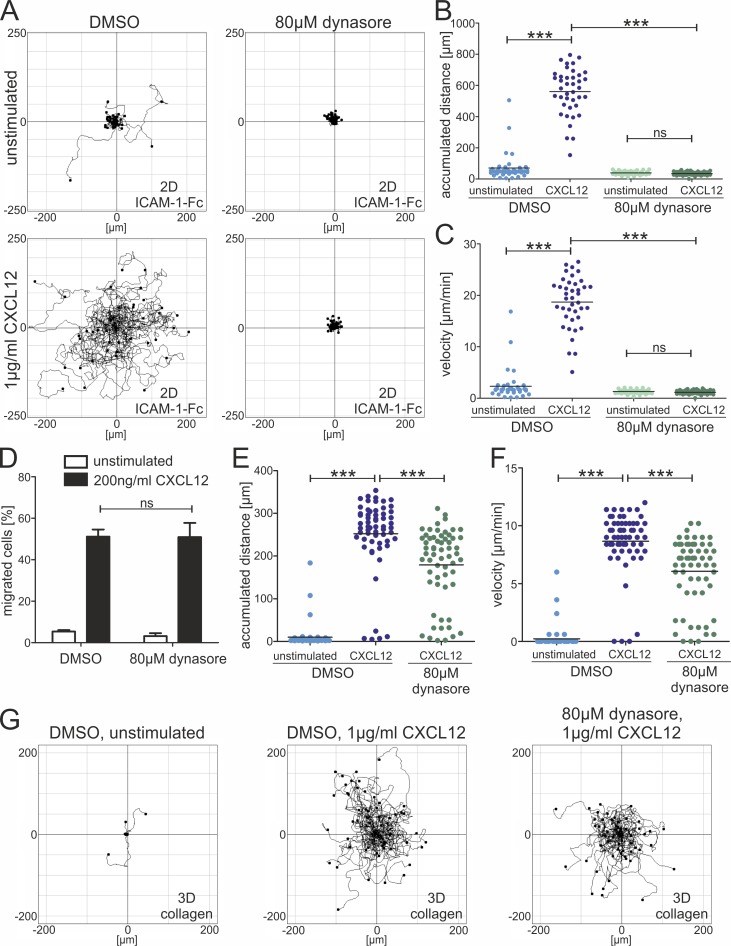
Adhesion-dependent T cell migration on 2D surfaces is regulated by dynamin2. (**A**) Migration tracks of primary human resting CD4^+^ T lymphocytes migrating on a 2D surface coated with ICAM-1-Fc for 30min. If indicated, migration was stimulated by adding 1μg/ml CXCL12 uniformly. Cells were incubated with either DMSO as a control or dynasore to inhibit dynamin2 activity. (**B**) Quantification of accumulated distance and (**C**) average migratory speed (velocity) of the migrating lymphocytes corresponding to (**A**). Results show one representative experiment out of three. (**D**) Quantification of chemotaxis of human resting CD4^+^ T cells through a polycarbonate transwell filter (3μm pore size) 4h after cells were seeded. If indicated, chemotaxis was stimulated by adding 200ng/ml CXCL12 to the lower well. Mean +SEM, n = 3. (**E**) Quantification of accumulated distance and (**F**) velocity of migrating human resting CD4^+^ T cells in a 3D collagen gel corresponding to (**G**). (**G**) Migration tracks of primary human resting CD4^+^ T lymphocytes migrating within a 3D collagen gel for 30min. If indicated, migration was stimulated by adding 1μg/ml CXCL12 uniformly. Results show one representative experiment out of three. ***P≤0.001, ns means not significant.

To analyze whether dynamin2 activity also influences lymphocyte motility, CXCL12-induced chemokinesis of CD4^+^ T cells on two-dimensional surfaces coated with integrin ligands was monitored. Migration of dynasore-treated lymphocytes was completely abolished compared to control cells (Fig 3A–3C; S3A–S3C Fig; S3 and S4 Videos). Cell migration on two-dimensional surfaces is strongly dependent on adhesion [43]. Since we did not observe an abrogation of the general chemotactic response of dynasore-treated lymphocytes to CXCL12 (Fig 3D), we analyzed if reduced motility might be a consequence of adhesion deficiencies. We therefore applied a three-dimensional collagen gel suitable to study integrin-independent cell migration [44,45]. In fact, we observed a strong CXCL12-induced migration of dynasore-treated T cells, although their velocity was decreased compared to control cells (Fig 3E–3G; S5 Video). Taken together, these findings suggest that dynamin2 activity is essential for adhesion-dependent migration of T lymphocytes but only marginally affects adhesion-independent migration.

### Chemical inhibition of vesicular trafficking and F-actin dynamics do not impair static lymphocyte adhesion

**Fig 4:**
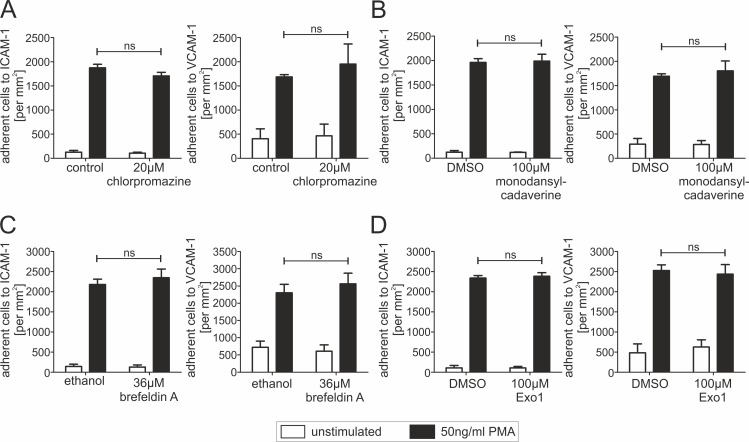
Different endo- and exocytosis inhibitors do not affect adhesion properties of human T lymphocytes. (**A**-**D**) Analysis of static adhesion of primary human resting CD4^+^ T cells to either ICAM-1-Fc or VCAM-1-Fc following treatment of the cells with different endo- or exocytosis inhibitors. If indicated, cells were stimulated with 50ng/ml PMA. 45min after seeding, the total number of adherent cells per mm^2^ was quantified. Endocytosis was inhibited by adding (**A**, n = 3) chlorpromazine or (**B**, n = 3) monodansylcadaverine. Exocytosis was inhibited by adding (**C**, n = 3) brefeldin A or (**D**, n = 3) Exo1. Mean +SEM, ns means not significant.

As dynamin2 plays a crucial role in endocytosis [22], we analyzed whether our observations might be due to defects in vesicular trafficking. Therefore, we treated human CD4^+^ T cells with dynamin2-independent inhibitors for endocytosis (chlorpromazine and monodansylcadaverine) and exocytosis (brefeldin A and Exo1). Strikingly, none of these inhibitors affected static CD4^+^ T lymphocyte adhesion (Fig 4), suggesting that adhesion defects observed in lymphocytes lacking dynamin2 activity are not due to impaired vesicular trafficking.

**Fig 5:**
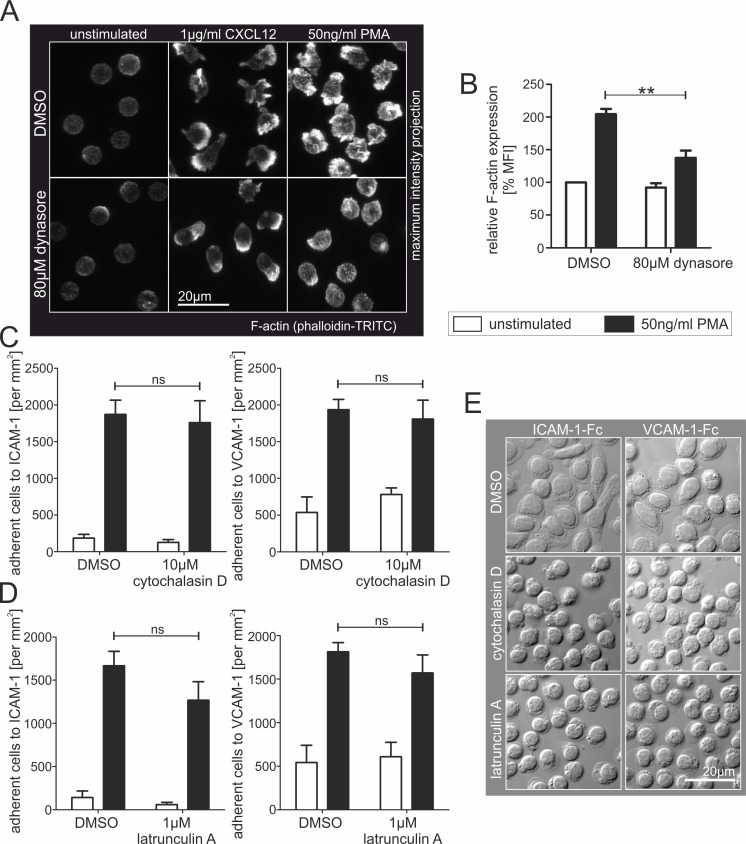
Dynamin2 partially regulates F-actin polymerization, which is dispensable for static adhesion of human resting CD4^+^ T cells. (**A**) Maximum intensity projections of confocal images from Z-stacks (0.3μm interval) of phalloidin-TRITC labeled resting human CD4^+^ T cells in suspension. If indicated, F-actin polymerization and/or cell polarization were stimulated by adding 1μg/ml CXCL12 or 50ng/ml PMA. (**B**, n = 5) FACS analysis of F-actin expression in primary resting human CD4^+^ T cells in suspension. Lymphocytes were either treated with DMSO as a control or with dynasore to inhibit dynamin2 activity. If indicated, actin polymerization was stimulated by adding 50ng/ml PMA for 45min. F-actin was specifically labeled by adding phalloidin-TRITC. (**C**, **D**) Analysis of static adhesion of primary human resting CD4^+^ T cells to either ICAM-1-Fc or VCAM-1-Fc following treatment of the cells with different F-actin polymerization inhibitors. If indicated, cells were stimulated with 50ng/ml PMA. 45min after seeding, the total number of adherent cells per mm^2^ was quantified. F-actin polymerization was inhibited with (**C**, n = 4) cytochalasin D or (**D**, n = 3) latrunculin A. (**E**) Detailed DIC images of representative ICAM-1-Fc or VCAM-1-Fc coated areas with adherent CD4^+^ T lymphocytes following treatment with F-actin polymerization inhibitors and stimulation with PMA. Mean +SEM, **P≤0.01, ns means not significant.

Several studies describe a role of dynamin2 in the regulation of F-actin polymerization [36,46]. Using confocal laser scanning microscopy we found that CXCL12- and PMA-stimulated F-actin polymerization observed in control T lymphocytes were strongly reduced in dynasore-treated cells accompanied by a shrunken leading edge, undersized pseudopodia and a missing uropod (Fig 5A). Accordingly, we confirmed diminished F-actin polymerization in dynasore-treated T cells by flow cytometry (Fig 5B). To elucidate if an abrogated F-actin polymerization causes lymphocyte adhesion deficiencies, we performed static adhesion assays using CD4^+^ T cells incubated with potent inhibitors of F-actin polymerization, cytochalasin D and latrunculin A. Cellular spreading, but not adhesion to integrin-ligands, was strongly abolished by these toxins (Fig 5C–5E). These findings suggest that the adhesion defects observed in lymphocytes lacking dynamin2 activity are not the consequence of impaired F-actin polymerization.

### Dynamin2 is essential for Rap1 activation

Vesicular trafficking influences several signaling processes, so we wondered if key signaling pathways would be affected by inhibition of dynamin2. Surprisingly, PMA-induced activation of Erk1/2 and Akt was not altered between DMSO- and dynasore-treated CD4^+^ lymphocytes, independent of whether integrin ligands were provided or not (S4A Fig).

**Fig 6:**
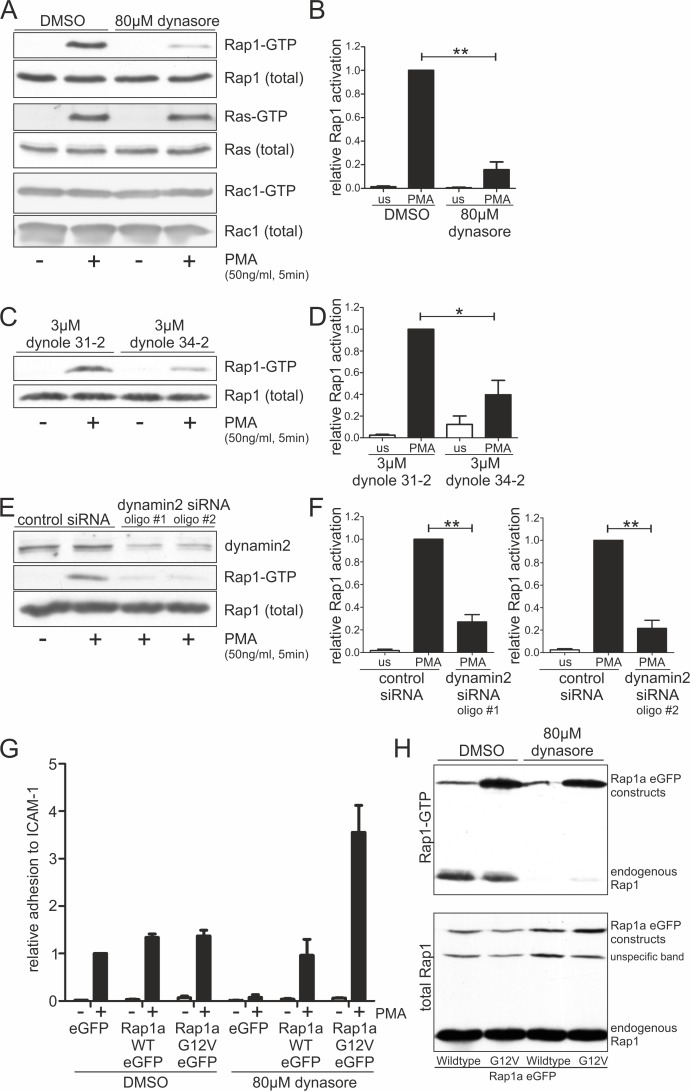
Dynamin2 is essential for Rap1 activation, a crucial step in T lymphocyte adhesion. (**A**-**F**) Western blot analyses of biochemical pull-downs of activated GTP-bound Rap1 (and Ras-GTP in (**A**)) via binding to immobilized GST-RalGDS-RBD (Rac1-GTP via GST-Pak1-PBD). Lysates were generated from primary human resting CD4^+^ T cells in suspension treated with (**A**) DMSO as a control or dynasore to inhibit dynamin2 activity, (**C**) dynole 31–2 as a control or dynole 34–2 to inhibit dynamin2 activity or (**E**) control siRNA or dynamin2 siRNA to knock down dynamin2 expression. If indicated, cells were stimulated by adding 50ng/ml PMA for 5min. (**B**, **D**, **F**) Quantifications of GTP-bound Rap1 corresponding to (**A**), (**C**) and (**E**), n = 3–4. (**G**, **H**) Analysis of primary human CD4^+^ T cells (**G**) or Jurkat E6.1 T cells (**H**) overexpressing either eGFP as a control, human Rap1a wildtype eGFP or a constitutively active Rap1a point mutant, Rap1a G12V eGFP. (**G**, n = 2) The relative static adhesion to ICAM-1-Fc following treatment of the cells with DMSO or dynasore was examined. PMA-stimulated adhesion of DMSO-treated eGFP-overexpressing primary CD4^+^ T cells was set to one. (**H**) Activation of endogenously as well as overexpressed Rap1 was analyzed in Jurkat E6.1 T cells applying a biochemical pull-down via GST-Ral-GDS-RBD and western blotting. Mean +SEM, *P≤0.05, **P≤0.01.

The small Ras-like GTPase Rap1 is an important regulator of lymphocyte polarization and adhesion [15,47,48]. We observed a strong abrogation of PMA-induced Rap1-activation, indicated by decreased amounts of Rap1-GTP found in a pull-down assay, in CD4^+^ T cells following treatment with dynasore, dynole 34–2 or RNAi of dynamin2 (Fig 6A–6F). Interestingly, activation of the small GTPases Ras and Rac1 was not affected by inhibition of dynamin2 activity (Fig 6A). We found that also TCR-signaling-dependent GTP-loading of Rap1 was strongly diminished in dynasore-treated CD4^+^ T cells (S4B Fig). Moreover, the high basal activity of Rap1 in CD4^+^ effector T cells [49] was sensitive to dynamin2 inhibition, suggesting that the perpetuation of Rap1 activity without direct external stimuli also depends on the large GTPase (S4C Fig). In line with this finding, dynasore treatment abrogated unstimulated integrin-dependent adhesion and migration of CD4^+^ effector T cells (S2C and S2D Fig; S4D–S4I Fig).

Although the activation of Rap1 strongly depends on dynamin2 activity, it is still unknown whether this is the reason for the adhesion deficiency observed in lymphocytes lacking dynamin2 function. We overexpressed eGFP fusion proteins of wildtype Rap1a and the constitutively active point mutant Rap1a G12V in primary human CD4^+^ T cells and performed static adhesion assays. As expected, dynasore-treated eGFP-overexpressing T cells displayed strong cell adhesion deficiencies in contrast to DMSO-treated control cells. This loss of adhesion could be rescued by overexpression of wildtype Rap1a eGFP or Rap1a G12V eGFP (Fig 6G). This is consistent with the finding that in Jurkat E6.1 T cells, the overexpressed Rap1a eGFP fusion proteins both were found in a GTP-bound state despite the presence of dynasore, while the activation of endogenously expressed Rap1 still was dependent on dynamin2 (Fig 6H).

Taken together, these data show that dynamin2 regulates the activation of the small GTPase Rap1 and thereby has a strong impact on T lymphocyte adhesion.

### Dynamin2 regulates T cell adhesion via FAK/Pyk2 and RapGEF1 activation

**Fig 7:**
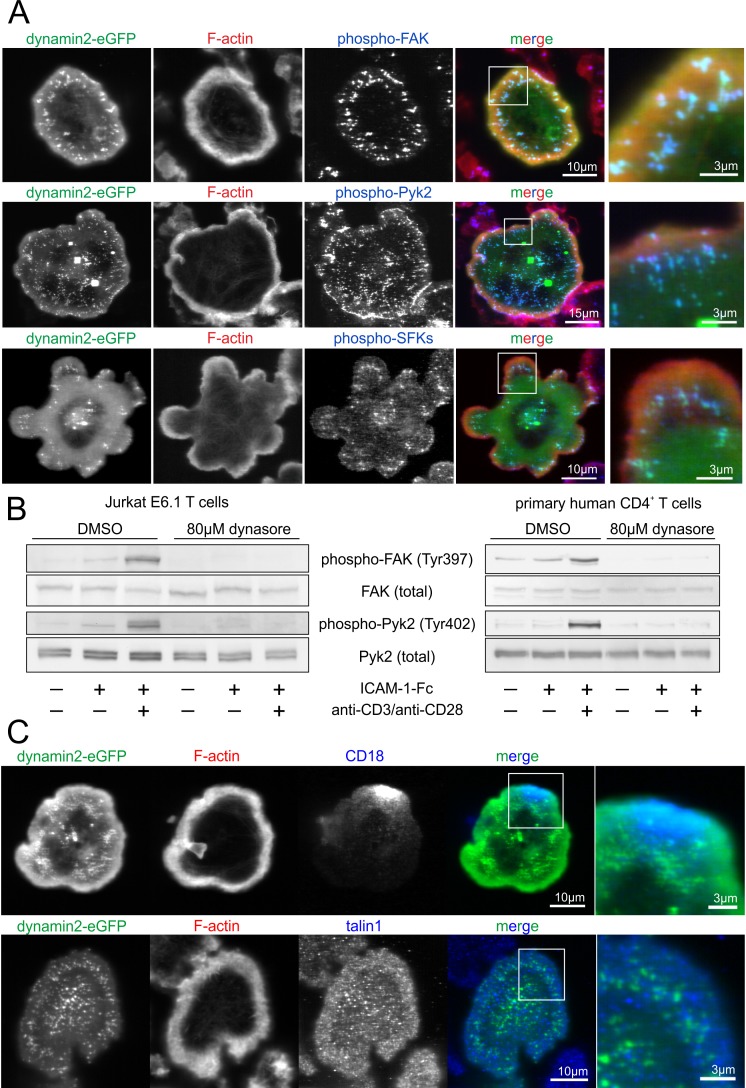
Dynamin2 eGFP colocalizes with and regulates the activity of different signaling molecules at the basal plasma membrane. (**A**,**C**) Dynamin2 eGFP was overexpressed in Jurkat E6.1 T cells. Cells were seeded on a surface coated with ICAM-1-Fc and activating anti-CD3 (10μg/ml) and anti-CD28 (20μg/ml) antibodies for 15min. After fixation, F-actin was labeled using TRITC-phalloidin and immunofluorescence staining of the following proteins was performed: phosphorylated focal adhesion kinase (FAK, Tyr397), phosphorylated protein tyrosine kinase 2 (Pyk2, Tyr402), phosphorylated SRC familiy kinases (SFKs, Tyr416 or equivalent), CD18, talin1. Images were acquired using a confocal laser scanning microscope with focus on the basal plasma membrane. (**B**) Western blot analysis of Jurkat E6.1 T cells or primary human CD4^+^ T cells which were either treated with dynasore to inhibit dynamin2 activity or with DMSO as a solvent control. Cells were seeded on either uncoated, ICAM-1-Fc-coated or ICAM-1-Fc/anti-CD3/anti-CD28-coated surfaces for 15min. Phosphorylation of FAK and Pyk2 were analyzed.

Focal adhesion kinase (FAK) and protein tyrosine kinase 2 (Pyk2) are known regulators of T cell adhesion and integrin activation. Upon autophosphorylation, they directly interact with SRC family kinases (SFKs) [50]. Immunofluorescent labeling of phosphorylated and therefore activated FAK, Pyk2 and SFKs revealed a strong co-localization of these proteins with dynamin2 eGFP in cluster-like structures at the basal plasma membrane of adherent T cells (Fig 7A). In addition, phosphorylation of FAK and Pyk2 was strongly dependent on dynamin2 activity, as it was not observed following dynasore treatment (Fig 7B).

Surprisingly, immunofluorescent labeling of CD18 or talin1 revealed that these proteins are not present in the dynamin2-enriched structures (Fig 7C), indicating that these are not directly linked to adhesion complexes but rather could serve as signaling complexes in adherent T cells.

**Fig 8:**
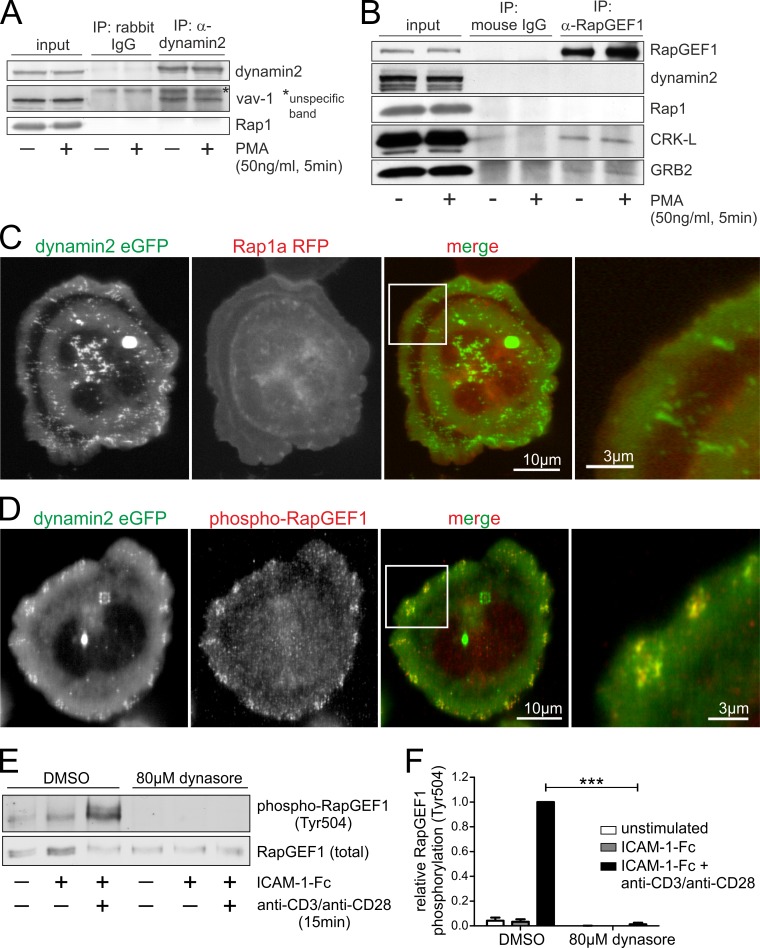
There is no evidence for a direct interaction of dynamin2 and Rap1 but dynamin2 colocalizes with phosphorylated RapGEF1 and regulates its activity. (**A-B**) Western blot analyses of immunoprecipitations using either anti-dynamin2 (**A**) or anti-RapGEF1 (**B**) antibodies and their respective isotype controls coupled to Protein G-coated dynabeads. Resting human primary CD4^+^ T cells were stimulated with 50ng/ml PMA if indicated. (**C-D**) Dynamin2 eGFP (in (**C**) Rap1a RFP as well) was overexpressed in Jurkat E6.1 T cells. Cells were seeded on a surface coated with ICAM-1-Fc and activating anti-CD3 (10μg/ml) and anti-CD28 (20μg/ml) antibodies for 15min. After fixation, immunofluorescence staining of phosphorylated RapGEF1 (Tyr504) was performed (**D**). Images were acquired using a confocal laser scanning microscope with focus on the basal plasma membrane. (**E**) Western blot analysis of primary human CD4^+^ T cells either treated with dynasore to inhibit dynamin2 activity or with DMSO as a solvent control. Cells were seeded on either uncoated, ICAM-1-Fc-coated or ICAM-1-Fc/anti-CD3/anti-CD28-coated surfaces for 15min. Phosphorylation status of RapGEF1 was analyzed. (**F**) Quantification of (**E**) with n = 4. Mean +SEM, ***P≤0.001.

We already proved that dynamin2 strongly regulates the activation of Rap1 (Fig 6A–6F), so we wondered whether the two proteins interact directly with each other. Interestingly, we were not able to co-immunoprecipitate Rap1 with dynamin2, although we observed a strong interaction of dynamin2 with vav1, a known binding partner of the large GTPase (Fig 8A). In addition, co-immunoprecipitation experiments with RapGEF1, an important guanine nucleotide exchange factor (GEF) for Rap1, also revealed no direct interaction between the GEF and dynamin2 or Rap1. However, we found a direct interaction between RapGEF1 and the adaptor molecules CRK-L and Grb2, known binding partners of dynamin2 as well as of RapGEF1 (Fig 8B).

Even if proteins do not directly interact with each other, they still might be located to the same cellular compartments. However, co-overexpression of dynamin2 eGFP and Rap1a RFP did not reveal a co-localization of the two proteins in adherent T lymphocytes (Fig 8C). In contrast, phosphorylated RapGEF1 strongly co-localized with dynamin2 eGFP in the cluster-like structures at the basal plasma membrane of adherent T cells (Fig 8D). The phosphorylation of RapGEF1 at tyrosine residue 504 is mediated by SFKs and known to activate its GEF activity for Rap1 [51,52]. We wondered whether chemical inhibition of dynamin2 might affect the phosphorylation of RapGEF1. Indeed, western blot analysis revealed that in human CD4^+^ T cells, RapGEF1 phosphorylation is strongly dependent on the activity of dynamin2 (Fig 8E and 8F).

These results suggest that dynamin2 is part of a scaffolding protein complex at the basal plasma membrane of adherent T lymphocytes and controls the phosphorylation and activation of RapGEF1 mediated by SFKs via FAK/Pyk2 signaling.

### Dynamin2 is essential for integrin valency regulation, but does not influence integrin affinity

**Fig 9:**
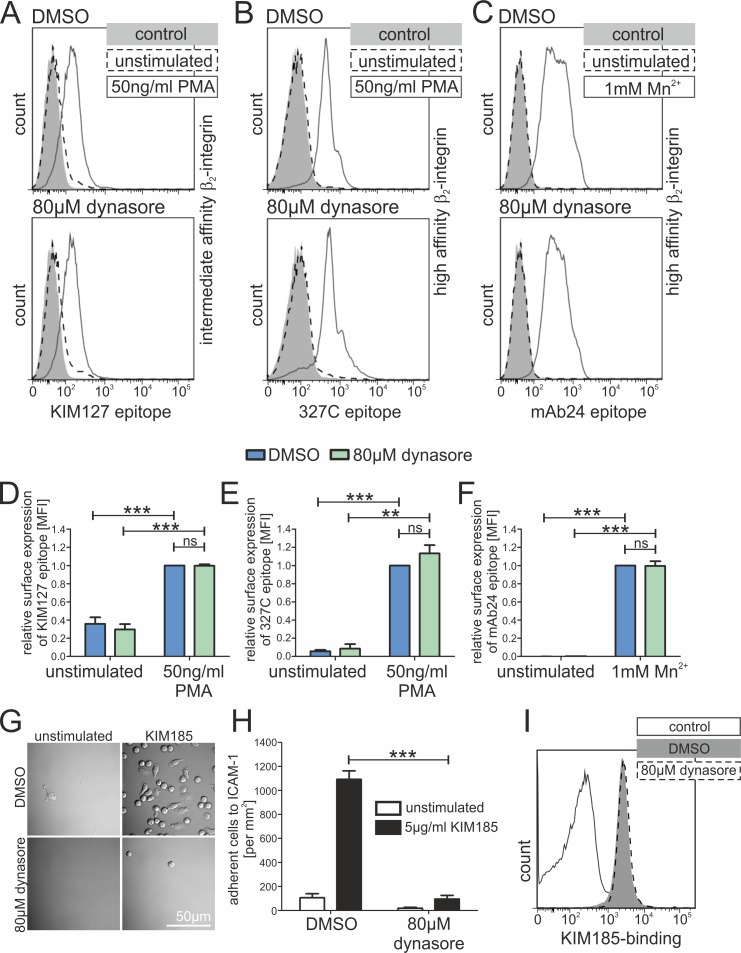
Chemical inhibition of dynamin2 does not affect integrin affinity regulation. (**A**-**F**) FACS analysis of the expression of different beta_2_-integrin (CD18) affinity states on the surface of primary human resting CD4^+^ T lymphocytes in suspension using specific monoclonal antibodies. Cells were either incubated with DMSO as a control or with dynasore to inhibit dynamin2 activity. If indicated, lymphocytes were stimulated with 50ng/ml PMA or 1mM Mn^2+^. Antibodies used were (**A**, **D**, n = 6) KIM127, recognizing intermediate affinity CD18, (**B**, **E**, n = 4) 327C and (**C**, **F**, n = 6) mAb24, both recognizing high affinity CD18. One representative histogram (**A**-**C**) as well as the relative quantification of surface expression with the stimulated DMSO sample set to 1 (**D**-**F**) is depicted for each antibody. (**G**, **H**) Analysis of the static adhesion of primary human resting CD4^+^ T cells to an ICAM-1-Fc coated surface. If indicated, adhesion was stimulated with KIM185, an antibody mechanically inducing the high affinity conformation of CD18 by binding it on the cellular surface. (**G**) Detailed DIC-images showing representative ICAM-1-Fc coated areas with adherent CD4^+^ T lymphocytes following incubation with DMSO/dynasore. (**H**, n = 4) Quantification of total adherent CD4^+^ T cells per mm^2^ 45min after seeding them on an ICAM-1-Fc coated surface. (**I**) Histogram depicting the results of a FACS analysis of the binding of KIM185 antibody to CD18 expressed on the surface of human resting CD4^+^ T cells following 2h of incubation with DMSO or dynasore. Mean +SEM, **P≤0.01, ***P≤0.001, ns means not significant.

Rap1 is a known regulator of integrin activity and was reported to be able to influence both, integrin affinity as well as valency. Having shown that dynamin2 controls Rap1 activation, we wondered whether integrin biology is altered in cells lacking dynamin2 activity. Interestingly, both resting as well as activated human CD4^+^ T cells treated with dynasore did not show an altered surface expression of important integrins compared to control cells (S5A and S5B Fig). Thus, we assessed integrin affinity regulation by applying monoclonal reporter antibodies which only bind to specific activation epitopes of integrins. Interestingly, we did not detect an altered expression of the intermediate (KIM127) or high (327C, mAb24) affinity conformations of beta_2_-integrins in dynasore-treated cells using flow cytometry (Fig 9A–9F) [53–55]. The same was observed for high affinity beta_1_-integrins (HUTS-4, S5C Fig) [56]. These findings were unexpected as adhesion of dynasore-treated cells is strongly affected, and we wondered whether the mere expression of a high affinity beta_2_-integrin is enough to induce adhesion in T cells. Therefore, we applied the monoclonal antibody KIM185, which directly binds CD18 and forces beta_2_-integrins into the high affinity conformation [57]. Static adhesion assays of resting CD4^+^ T cells revealed that control but not dynasore-treated lymphocytes nicely adhered to ICAM-1 after stimulation with KIM185 (Fig 9G and 9H), although binding efficiencies of the antibody were not changed (Fig 9I).

**Fig 10:**
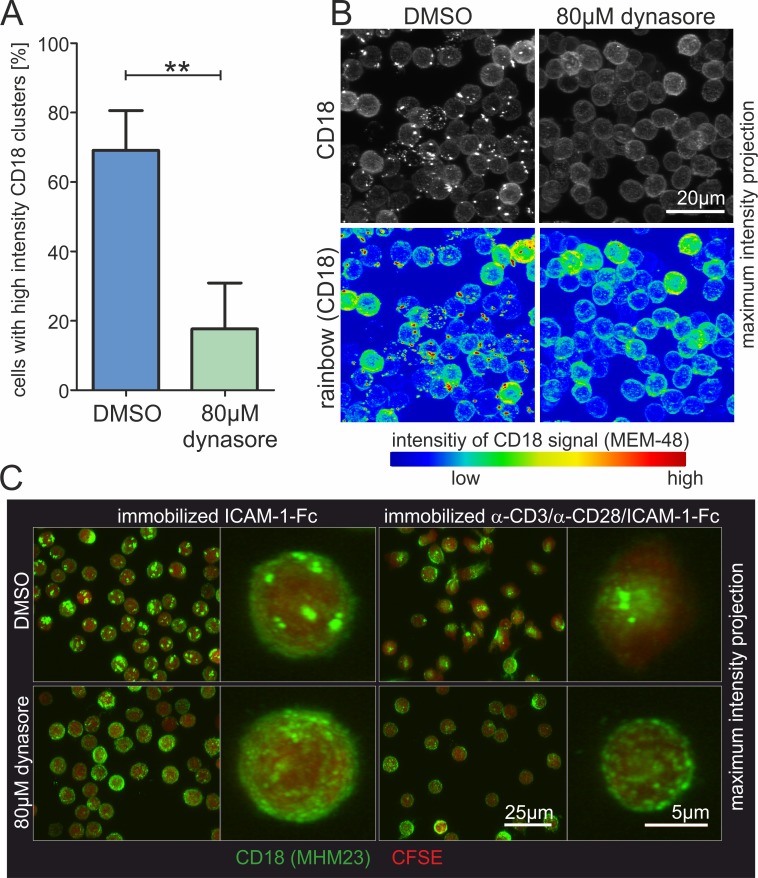
Integrin clustering strongly depends on dynamin2 in human CD4^+^ T cells. (**A**, **B**) Analysis of beta_2_-integrin clustering on primary human resting CD4^+^ T lymphocytes in suspension. Cells were incubated for 2h with either DMSO as a control or dynasore to inhibit dynamin2 activity. Subsequently, cells were fixed with paraformaldehyde and the beta_2_-integrin chain was immunofluorescently labeled using the monoclonal antibody MEM-48. Lymphocytes were seeded in microchannels and after settlement Z-stack images were acquired using a confocal laser scanning microscope. (**A**, n = 4) T cells bearing prominent “high intensity” beta_2_-integrin clusters were quantified. (**B**) Representative maximum intensity projections of Z-stacks (0.3μm interval) depicting T lymphocytes in suspension following staining of beta_2_-integrin. (**C**) Analysis of beta_2_-integrin clustering on primary human resting CD4^+^ T lymphocytes exposed to coated surfaces. First, cells were covalently labeled with the cell tracer CFSE. Subsequently, they were incubated for 1h 45min with either DMSO as a control or dynasore to inhibit dynamin2 activity. Then beta_2_-integrins were stained using the monoclonal antibody MHM23. The lymphocytes were seeded for 45min on surfaces coated with either ICAM-1-Fc alone or a mixture of ICAM-1-Fc and anti-CD3/CD28 antibodies to mimic an antigen presenting cell. Finally, Z-stacks (0.3μm interval) were acquired using a confocal laser scanning microscope. Mean + SEM, **P≤0.01.

We then analyzed whether integrin valency regulation might be influenced in T cells deficient for dynamin2 activity. Immunofluorescent labeling of beta_2_-integrin on unstimulated T cells in suspension revealed a strong clustering of the integrin into many macroclusters on the cell surface which was completely absent in dynasore-treated lymphocytes (Fig 10A and 10B). Furthermore, the massive clustering of CD18 into one single area at the basal plasma membrane in T cells seeded on a surface coated with anti-CD3/CD28 antibodies and ICAM-1-Fc was totally abrogated in lymphocytes without dynamin2 activity (Fig 10C). We also observed a clustering of the CD18 high affinity stimulating antibody KIM185 at the uropod of polarized T cells, a structure that was mainly missing in cells treated with dynasore (Fig 5A; S6A Fig). In addition, KIM185-treated control T cells seeded on ICAM-1-Fc showed a strong clustering of the labeled beta_2_-integrin, which was not observed in T cells deficient for dynamin2 activity (S6B Fig; S6 Video).

Taken together, these results suggest an important role of dynamin2 in integrin valency but not affinity regulation in T lymphocytes.

## Discussion

Our work shows that dynamin2 is a critical component of integrin valency regulation in CD4^+^ T cells and provides a detailed analysis of its role in adhesion regulation of human lymphocytes. Both chemical inhibition of dynamin2 activity and RNAi of dynamin2 result in dramatic loss of integrin-mediated adhesion and migration of human T cells. Our results further suggest that dynamin2 organizes Rap1 signaling towards integrins via the localized activation of RapGEF1. Surprisingly, dynamin2-mediated Rap1 activation does not involve intra-molecular affinity modulation of cell surface integrin molecules in human CD4^+^ T cells. This novel regulatory link that exists between dynamin2 and Rap1 signaling appears to selectively target mechanisms of clustering in integrin activation.

The highly conserved role of dynamin proteins in the endocytosis of cell surface receptors is well understood at the genetic, molecular and structural levels [22]. Furthermore, dynamin2 was shown to be essential for proper T cell recirculation and homing *in vivo* via sphingosine-1-phosphate receptor internalization [38]. The strong inhibition of human T cell adhesion to integrin ligands by the dynamin inhibitor dynasore (Fig 1) indicated a possible inhibition of endocytosis of relevant signaling receptors or the integrin molecules themselves. However, flow cytometry analyses revealed that integrin surface expression on CD4^+^ T cells is not affected by dynasore at steady state (S5 Fig). Furthermore, PMA-stimulated CD4^+^ T cell adhesion to integrin ligands, which is strongly abrogated by dynasore, is not affected by inhibitors of vesicular trafficking in the rather short time scale of our experimental system (<1hr), which renders the involvement of vesicle transport dynamics as a subcellular basis for our observations unlikely (Fig 4).

Dynamin2 has been implicated in T cell activation signaling via internalization of the T cell receptor, which may account for its sustained signaling from intracellular locations [37], or via actin cytoskeletal reorganization at the immunological synapse [36]. However, our observations on the dynamin2 involvement in human CD4^+^ T cell adhesion are made on the scale of minutes post stimulation, which makes a contribution of sustained TCR signaling to these processes unlikely. We furthermore observe a strong role of dynamin2 in chemokine induced integrin-dependent T cell adhesion and migration (Figs 1–3; S3 Fig), which both depend on heterotrimeric G protein signaling, and are therefore unrelated to TCR-mediated events. However, we cannot fully rule out an influence of TCR internalization on integrin inside-out signaling in long-term *in vivo* processes, e.g. during antigen presentation. Furthermore, we observe a moderate effect of dynasore on actin polymerization in CD4^+^ T cells (Fig 5). On a similar issue, dynasore has been shown by others to affect the actin cytoskeleton, and that this perturbation could still be observed in dynamin triple knockout cells and is thus partially dynamin-independent [58]. However, we rule out a strong contribution of actin to our system of lymphocyte adhesion, since integrin-dependent adhesion of rounded cells is still strongly stimulated by PMA when potent inhibitors of actin polymerization are used (Fig 5). Furthermore, we have reproduced our key observations by making use of the alternative dynamin inhibitor dynole 34–2, or by RNAi of dynamin2 (Figs 1 and 2).

The small GTPase Rap1 has been shown to be essential for integrin-mediated lymphocyte adhesion [48]. Our data clearly show that the activation of Rap1 depends on dynamin2 (Fig 6). Defective GTP loading of endogenous Rap1 is the explanation for the loss of adhesion in lymphocytes lacking dynamin2 activity, as overexpression of Rap1a constructs rescues this phenotype.

It has been reported that in strongly adherent cells dynamin2 and Src family kinases (SFKs) interact directly with FAK and Pyk2 to form signaling complexes [30,59]. This is in line with our finding that these proteins strongly co-localize in cluster-like structures at the basal plasma membrane of adherent T cells following TCR-stimulation. The autophosphorylation of FAK and Pyk2 is a prerequisite for those interactions to occur, and is strongly dependent on dynamin2 (Fig 7). The absence of CD18 and talin1 from these complexes suggest that they are not adhesion sites targeted for internalization but rather signaling platforms, which also include phosphorylated RapGEF1 (Figs 7 and 8). We observed a direct interaction of RapGEF1 with the adaptor proteins CrkL and GRB2, both were previously reported to mediate RapGEF1 membrane recruitment [60]. Phosphorylation at Tyr504 of RapGEF1 is mediated by SFKs and activates its GEF function for Rap1 [52,61]. As we found the activation of RapGEF1 to be strongly dependent on dynamin2 as well (Fig 8), we suggest that dynamin2 is important for the formation of the signaling complexes found at the basal plasma membrane of T cells by modulating FAK/Pyk2 signaling, thereby mediating the SFK-induced phosphorylation of RapGEF1 and, ultimately, the activation of Rap1. In addition, this could result in a positive feedback loop, as it was reported before that not only Src/FAK/Pyk2 are important for Rap1-activation [62–64], but also that Rap1-GTP controls the activation of FAK/Pyk2 [65,66].

The precise mechanisms of integrin activation have been subjects to intense studies in various experimental systems [1,5,67]. While it is clear that talin or the kindlins are involved in conformational regulation of the integrins [68–70], the precise role of Rap1-mediated integrin activation remains elusive. Several studies describe a role of Rap1 in integrin affinity [66,71–73], others in integrin affinity as well as valency [74,75] and again others only in integrin valency regulation [15,16,76]. However, several downstream effectors of Rap1 with different effects on integrins exist [77]. For example, RIAM has been shown to be important for talin recruitment to the platelet integrin alpha_IIb_beta_3_ and its activation *in vitro* [78–80]. Surprisingly, platelets from RIAM-null mice showed normal integrin functions, suggesting that RIAM-independent mechanisms of integrin activation exist [81]. Nevertheless, two recent studies prove the importance of RIAM for integrin-dependent leukocyte trafficking *in vivo* [82,83]. RapL is another important Rap1 effector enriched in lymphoid tissues. The Rap1-GTP-RapL signaling axis has been shown to regulate integrin clustering and polarization [84–87], illustrating that Rap1 has different possibilities to achieve integrin activation depending on the respective signaling route and interaction partners.

Surprisingly, we found no evidence for a perturbation of high affinity epitope detection on both beta_1_- and beta_2_-integrins following dynasore treatment (Fig 9; S5 Fig). This is in line with previous findings reporting that the stimulation of integrin activation epitopes is not necessarily Rap1-dependent in all cellular models [88]. In addition, T cell adhesion stimulated by KIM185, an antibody mechanically forcing beta_2_-integrins into high affinity conformation [57], was sensitive to inhibition of dynamin2 activity (Fig 9). Accordingly, KIM185-mediated lymphocyte adhesion was previously reported to be dependent on Rap1 [89].

Several of our experimental results point to the involvement of dynamin2 in the much less well understood valency regulation of integrins [90]. Already at steady state, we observe a strong reduction of CD18-positive clusters at the cell surface of CD4^+^ T cells following inhibition of dynamin2 activity, and dynasore further inhibits the redistribution of beta_2_-integrin clusters by immobilized anti-CD3/CD28 antibodies (Fig 10). Finally, we also observed clustering of KIM185-induced high affinity beta_2_-integrins, which was also sensitive to dynamin 2 inhibition (S6 Fig).

In summary, our data provide insight into valency regulation of integrins *in vitro*, most notably identifying dynamin2 as a novel regulator of T lymphocyte adhesion via FAK/Pyk2- and RapGEF1-mediated Rap1 activation. Our findings suggest that dynamin2 acts, independent of its established function in endocytosis, as part of a scaffold protein complex to support integrin clustering.

## Material and methods

### Antibodies

The following antibodies were used for protein detection and/or immunoprecipitation: anti-AKT (#9272, Cell Signaling), anti-phospho-AKT (#4060, Cell Signaling), anti-CD3 (clone OKT3, purified from hybridoma supernatant), anti-CD11a (clone MHM24, Dako), anti-CD18 (clone MHM23, Dako; clone MEM-48, Exbio; #bs-0503R, BIOSS), anti-CD28 (clone CD28.05, BD), anti-CD29 (clone MEM101A, Immunotools; clone 12G10, Abcam), anti-CD49d (clone 9F10, eBioscience), anti-CD49e (clone SAM-1, Abcam), anti-CD49f (clone GoH3, BD), anti-CRKL (sc-319, Santa Cruz), anti-dynamin2 (ab3457, Abcam), anti-Erk1/2 (#9102, Cell Signaling), anti-phospho-Erk1/2 (#9101, Cell Signaling), anti-human IgG Fc (#109-005-098, Jackson), anti-FAK (#3285, Cell Signaling), anti-phospho-FAK (#3283, Cell Signaling), anti-GRB2 (sc-255, Santa Cruz), anti-integrin beta_7_ (clone FIB504, BD), anti-Pyk2 (#3480, Cell Signaling), anti-phospho-Pyk2 (#3291, Cell Signaling), rabbit IgG control (#12–370, Millipore), anti-Rac1 (clone 102/Rac1, BD), anti-Rap1 (#16120, Thermo Scientific), anti-RapGEF1 (sc-17840, Santa Cruz), anti-phospho-RapGEF1 (#A1120, Assay Bio Tech), anti-Ras (clone Ras10, Millipore), anti-phospho-Src family kinases (#2101, Cell Signaling), anti-talin1 (ab71333, Abcam), anti-vav (sc-132, Santa Cruz).

The following monoclonal antibodies were used to analyze or induce affinity-changes of integrins: clone KIM127 (CD18 intermediate affinity, kindly provided by Nancy Hogg), clone mAb24 (CD18 high affinity, kindly provided by Nancy Hogg), clone 327C (CD18 high affinity, kindly provided by Donald E. Staunton), clone KIM185 (CD18 high affinity inducing, purified from hybridoma supernatant), clone HUTS-4 (CD29 high affinity, Millipore).

### Cell isolation and culture

Primary human peripheral blood mononuclear cells (PBMCs) were prepared from standard buffy coat preparations of healthy blood donors using Ficoll (Pan Biotech) density gradient centrifugation. Subsequently, specific cell populations were isolated using an AutoMACS Pro S/N 614 device (Miltenyi) and, depending on cell type and assay, one of the following cell separation reagents (Miltenyi): CD4 Microbeads (130-045-101), CD4^+^ T cell Isolation Kit (130-096-533), CD8^+^ T cell Isolation Kit (130-096-495), NK cell Isolation Kit (130-092-657) and CD19 Microbeads (130-050-301). Cells were cultured at 37°C and 5% CO_2_ in VLE-RPMI-1640 medium (Biochrom), containing 10% fetal calf serum (Sigma), 100 U/ml Penicillin and 100 μg/ml Streptomycin (PAN Biotech). Buffy coats were received after written consent following protocols accepted by the institutional review board at the University of Bonn (local ethics vote number 203/09). For each sample an informed written consent was provided according to the Declaration of Helsinki.

Jurkat E6.1 T lymphocytes were purchased from ATCC (TIB-152) and cultured at 37°C and 5% CO_2_ in RPMI-1640 Medium (GE Healthcare), containing 10% fetal calf serum (Sigma) and 40μg/ml Gentamicin (PAN Biotech).

### Chemical inhibitors

The following small molecules were used for the chemical inhibition of specific proteins or cellular processes: brefeldin A (B6542, Sigma-Aldrich), chlorpromazine (C8138, Sigma-Aldrich), cytochalasin D (C8273, Sigma-Aldrich), dynasore hydrate (D7693, Sigma-Aldrich), dynole 34–2 (ab120463, Abcam), dynole 31–2 (ab120464, Abcam), Exo1 (#1850, Tocris), latrunculin A (L5163, Sigma-Aldrich), monodansylcadaverine (30432, Sigma-Aldrich). Except for Brefeldin A and Chlorpromazine, which were diluted in ethanol or water respectively, all stock solutions were prepared using dimethyl sulfoxide (DMSO, #276855, Sigma-Aldrich). Cells were preincubated at 37°C and 5% CO_2_ with the specific inhibitors diluted in HBSS for 60min (cytochalasin D, latrunculin A) to 100min (all others) and were present during all functional analyses.

### Cell transfection

Primary human CD4^+^ T cells were transfected directly after isolation using the Amaxa Nucleofector I device and the human T cell Nucleofector Kit (#VPA-1002, Lonza). Transfection was performed according to the instructions of the manufacturer (program V24, 5μg vector DNA or 300nM small interfering RNA (siRNA), respectively). Jurkat E6.1 T lymphocytes (1x10^7^ per sample) were transfected using the Gene Pulser X cell device equipped with a CE module (Bio-Rad) and 4mm cuvettes (Bridge). Transfection with 10–30μg plasmid DNA was carried out in 500μl RPMI-1640 (GE Healthcare) with 50% FCS (Sigma) using an exponential protocol (240V, 1500μF). Cells were used for functional assays 24h (transfection with DNA) or 48h (transfection with siRNA) later and cultured without antibiotics post-transfection. RNA interference was performed using oligonucleotides with the following sense sequences:

5’-GGUGCCUGUAGGUGAUCAA(dTdT)-3’ (human Dynamin2, oligo #1),

5’-GCACUCUGUAUUCUAUUAA(dTdT)-3’ (human Dynamin2, oligo #2),

5’-AAACAUGCAGAAAAUGCUG(dTdT)-3’ (Renilla luciferase, control siRNA).

All siRNAs were purchased from Quiagen or Dharmacon RNA Technologies, respectively.

### Small GTPase activation assays

Biochemical pull-down of GTP-bound small GTPases was performed using commercially available kits (Thermo Scientific, #16120 for Rap1 and Ras and #16119 for Rac1). Pull-down and detection of activated GTPases was performed according to the indications of the manufacturer. Briefly, GST-RalGDS-RBD (for Rap1 and Ras) or GST-Pak1-PBD (for Rac1) was immobilized on glutathione resin in spin cups. The cell lysate of interest was incubated in these spin cups for 1h at 4°C with gentle rocking. After three washing steps, bound protein was eluted from the glutathione resin and analyzed by standard SDS polyacrylamide gel electrophoresis and western blotting. Activation of small GTPases was analyzed using the gel analyzer in ImageJ.

### Static adhesion assay

Adhesion of immune cells under static conditions was analyzed using 35mm petri dishes coated with different integrin ligands (human ICAM-1-Fc (intercellular adhesion molecule 1) or VCAM-1-Fc (vascular cell adhesion molecule 1) immobilized with goat anti-human IgG Fc-specific antibody (Jackson) bound to plastic via hydrophobic interactions, human fibronectin (Harbor Bio-Products)) only in their middle part to provide uncoated border areas as control. Blocking of the hydrophobic surface was performed using 1% bovine serum albumin (Roth) in PBS (PAA). 2x10^6^ cells in 1ml HBSS (PAA) were seeded per dish and incubated at 37°C and 5% CO_2_ for 45min. When indicated, cell adhesion was stimulated using different stimuli (1μg/ml CXCL12 (Peprotech), 50ng/ml phorbol 12-myristate 13-acetate (PMA, Sigma), 5μg/ml KIM185 antibody, 2x10^6^ anti-CD3/CD28 coated dynabeads (OKT3, CD28.05, beads from life technologies)). Non-adherent cells were aspirated and the dishes were washed twice with prewarmed HBSS. Still adherent cells were fixed using 4% paraformaldehyde/HBSS and analyzed using a Nikon Eclipse TE2000 microscope equipped with a CCD 1300 Vosskühler camera and ImageJ.

### Adhesion assay under flow

*In vitro* analysis of adhesion under conditions of unidirectional laminar flow was carried out using a custom-built parallel-plate flow chamber. A mixture of the integrin ligands ICAM-1-Fc and VCAM-1-Fc was immobilized on a petri dish (see “static adhesion assay”) and covered with 1μg/ml human CXCL12 (Peprotech) for 15min at 37°C. The dish was assembled in the flow-chamber and the T cells (2x10^6^/ml in HBSS supplemented with 10mM Hepes) drawn through the chamber at a controlled flow rate with a syringe pump attached to the outlet. The unidirectional laminar flow resulted in a shear stress of 1dyn/cm^2^ or 2.8dyn/cm^2^ at the inner wall of the chamber, depending on the flow rate used in the assay. After 30min the quantity of T cells attached to the immobilized integrin ligands in the chamber was determined. Cells were analyzed using a Nikon Eclipse TE2000 microscope equipped with a CCD 1300 Vosskühler camera and ImageJ.

### Transwell migration assay

Chemotaxis of primary human CD4^+^ T cells was analyzed using transwell migration assays. 5x10^5^ T cells in 100μl VLE-RPMI-1640 with 0.5% FCS were placed to the upper compartment of uncoated polycarbonate filters (Costar, Corning, 3μm pore size). If stated, 200ng/ml CXCL12 (Peprotech) was added to the 600μl VLE-RPMI-1640 with 0.5% FCS in the lower compartment. Control assays were performed without chemokine. Transmigrated cells were counted after incubation of 4h at 37°C and 5% CO_2_.

### Analysis of two-dimensional lymphocyte chemokinesis

Migration of lymphocytes on two-dimensional surfaces was analyzed in μ-Slide I channels (Ibidi) to prevent artifacts generated by evaporation or drift. The slides were coated with a goat anti-human IgG Fc-specific antibody (Jackson) and subsequently were blocked with 1% BSA in PBS at 4°C over night. Human ICAM-1-Fc or VCAM-1-Fc was immobilized on the antibody coated surface (1h at room temperature). Slides were washed twice and pre-equilibrated at 37°C and 5% CO_2_. 4x10^5^ lymphocytes in 100μl HBSS were transferred to the channel and, if stated, chemokinesis was induced by adding 1μg/ml CXCL12 (Peprotech) uniformly. Live cell imaging was performed at 37°C for 30min using a fully automated inverted Nikon Eclipse TE2000 microscope equipped with a motorized xyz-stage (Märzhäuser) and a CCD 1300 Vosskühler camera. Migration parameters were calculated using the Manual Tracking and Chemotaxis Tool plugins in ImageJ.

### Analysis of lymphocyte chemokinesis in a three-dimensional collagen gel

To analyze lymphocyte migration in a three-dimensional collagen gel, collagen I (Pure Col, Advanced Biomatrix) was mixed with 7.5% sodium bicarbonate (Invitrogen) and 10x HBSS (Gibco) on ice. Lymphocytes in HBSS were carefully mixed with the collagen solution at a 2:1 ratio, resulting in gels with a collagen concentration of 1.6mg/ml. Collagen-lymphocyte mixtures were carefully placed in μ-Slide 8 Wells (Ibidi) and incubated for polymerization at 37°C and 5% CO_2_ for 30min. Afterwards, collagen gels including cells were covered with HBSS to prevent drying-out. If stated, 1μg/ml CXCL12, 80μM dynasore or DMSO were present in the collagen-lymphocyte mixtures and the covering HBSS. Live cell imaging was performed at 37°C for 30min using a fully automated inverted Nikon Eclipse TE2000 microscope equipped with a motorized xyz-stage (Märzhäuser) and a CCD 1300 Vosskühler camera. Migration parameters were calculated using the Manual Tracking and Chemotaxis Tool plugins in ImageJ.

### Analysis of integrin clustering

Clustering of beta_2_-integrins on primary resting human CD4^+^ T cells was analyzed with two different approaches. On the one hand, pre-ligand integrin clustering was analyzed using untouched and unstimulated T cells. 24h post isolation the cells were harvested for 2h in HBSS and fixed in suspension with 2% paraformaldehyde (PFA) in PBS for 10min at room temperature. 2% glycine in PBS was added for another 10min in a 1:1 ratio. Following a washing step the cells were stained with an anti-CD18 (MEM-48) and an anti-mouse Cy3 antibody (#115-166-062, Jackson). The lymphocytes were then seeded in μ-Slide VI 0.4 channels (Ibidi) and analyzed after settling using an inverted Zeiss 5 Live confocal laser scanning microscope and a Plan-Fluar 100x/1.45 oil immersion Objective (Carl Zeiss). Z-stacks with 0.3μm interval were taken and maximum Z-projections were generated. A rainbow filter was applied to the resulting image and cells were counted and divided into two different groups depending on whether they exhibited high intensity integrin clusters or not.

Post-ligand integrin clustering was analyzed using a live cell imaging approach. T cells were stained with 1μM CFSE (Invitrogen), an anti-CD18 (MHM23) and an anti-mouse Cy3 antibody (Jackson). Then they were resuspended in HBSS and seeded in μ-Slide I channels (Ibidi) covered with either anti-CD3 (OKT3, 2μg/ml) / anti-CD28 (CD28.05, 4μg/ml) antibodies and ICAM-1-Fc or only ICAM-1-Fc as a control. Cells were incubated at 37°C and 5% CO_2_ for 45min. Z-stacks were taken with an inverted Zeiss 5 Live confocal laser scanning microscope equipped with a climate chamber (37°C, 5% CO_2_, humidified) and a Plan-Fluar 100x/1.45 oil immersion Objective (Carl Zeiss).

### Live cell imaging

Jurkat E6.1 T cells overexpressing different GFP- and/or RFP-fusion proteins were analyzed using an inverted Zeiss 5 Live confocal laser scanning microscope equipped with a climate chamber (37°C, 5% CO_2_, humidified) and a Plan-Fluar 100x/1.45 oil immersion Objective (Carl Zeiss). Cells were resuspended in HBSS and seeded in μ-Slide I channels (Ibidi) to prevent artificial cell movement generated by evaporation or fluid-flow. Depending on the assay the inner surface of the slide was coated with different integrin ligands or stimulating antibodies (anti-CD3, anti-CD28) to mimic an antigen presenting cell.

### Differential Interference Contrast (DIC) microscopy

Primary human CD4^+^ T cells were seeded in μ-Dishes (Ibidi) coated with ICAM-1-Fc or VCAM-1-Fc (see “Static adhesion assay”). Adherent lymphocytes were analyzed using an inverted Olympus Fluoview 1000 confocal laser scanning microscope equipped with a Plan Apochromat 60x, NA 1.4 oil immersion objective (Olympus), a climate chamber (5% CO_2_, 37°C, humidified, Evotec) and DIC.

### Surface and intracellular flow cytometry

Most surface stainings were performed at 4°C for 20min except for integrin affinity reporter antibodies (KIM127, 327C, mAb24, HUTS-4), which were added to cells at 37°C for 20-30min. After incubation with the primary antibody, cells were washed with ice cold staining buffer and if necessary were incubated with a fluorescently labeled secondary antibody for 15min at 4°C. For intracellular staining of filamentous actin, cells were fixed with 2%PFA/PBS for 10min at room temperature. Subsequently, 2% glycine/PBS was added for 10min. Cells were permeabilized (0.1% saponin) for 10min at 4°C and then stained with phalloidin-Alexa488 (Sigma) for 20min at room temperature. Analysis was carried out using a FACS-Canto II flow cytometer (Becton Dickinson) and analyzed with FlowJo software (Treestar).

### Co-immunoprecipitation

Immunoprecipitation of specific proteins was performed using protein G coupled Dynabeads (Life Technologies). If necessary, 2μg of antibody was coupled to the beads for 1 hour at 4°C. Cells were lysed in Triton lysis buffer (10mM HEPES, 2mM MgCl_2_, 10mM KCl, 0.5mM EDTA, 150mM NaCl, 0.5% Triton) for 5min on ice and centrifuged at 16.000g for 5min. Supernatant protein concentrations were measured by standard BCA assay. At least 500μg of total protein was transferred to the beads and incubated at 4°C for 4h under constant movement. After three washing steps the remaining protein bound to the beads was eluted and analyzed with standard polyacrylamide gel electrophoresis and western blotting.

### Semi-quantitative real-time PCR

For cDNA synthesis, RNA was isolated from primary human CD4^+^ T cells using TRIzol (Invitrogen) according to the manufacturer’s instructions. Synthesis of cDNA was carried out with the High Capacity cDNA Reverse Transcription Kit (Applied Biosystems). Semi-quantitative PCR was carried out using an iCycler IQ5 (BioRad) and a Taqman Gene Expression Assay (Applied Biosystems) with the following probes: Hs00189369_m1 (Dynamin1), Hs00974698_m1 (Dynamin2) and Hs00399015_m1 (Dynamin3) as well as 4352934E (GAPDH) and 18S (Eurofins,

for: 5’-GATCCATTGGAGGGCAAGTCTG-3’,

rev: 5’-ACGAGCTTTTTAACTGCAGCAACTTTA-3’,

probe: 5’-CAGCCGCGGTAATTCCAGCTCCAATAGCGT-3’) as references.

### Statistical analysis

Statistical analysis was performed using Prism Software (Graph Pad). Depending on the assay, either paired or unpaired Student’s t-test was calculated.

## Supporting information

S1 Fig Human T cells are a good model system to study integrin-mediated adhesion and they strongly express dynamin2. (**A**) Scheme of the experimental setup to study integrin-mediated cell adhesion under static conditions. The center of a petri dish is coated with an integrin ligand whereas the periphery is not. Phase contrast images depict boundary areas between the coated and non-coated surfaces in the petri dish with PMA-stimulated, adherent primary human resting CD4^+^ T cells, which strongly depend on the presence of an integrin ligand to be able to adhere firmly to the surface. (**B**) Phase contrast images of human resting CD4^+^ T cells adherent to ICAM-1-Fc. The cells need to be stimulated (in this case with 50ng/ml PMA) in order to adhere to the provided integrin ligand. (**C**) Semi-quantitative PCR analysis of the mRNA expression of dynamin1, dynamin2 and dynamin3 in primary resting human CD4^+^ T cells. Mean +SEM, *** P≤0.001, n = 5. (**D**) Confocal images depicting a Jurkat E6.1 T cell overexpressing human dynamin2 eGFP and lifeact RFP. The cell is plated on a surface coated with ICAM-1-Fc as well as anti-CD3 (10μg/ml) and anti-CD28 (20μg/ml) antibodies. Focus on basal plasma membrane.
(PDF)

S2 Fig Integrin-mediated adhesion of human lymphocytes strongly depends on dynamin2 activity. (**A**, **C**-**J**) Analysis of the adhesion of different types of primary human lymphocytes to either fibronectin, ICAM-1-Fc or VCAM-1-Fc under static conditions. Lymphocytes were treated with DMSO as a control or 80μM dynasore to inhibit dynamin2 activity. If indicated, adhesion was stimulated with 50ng/ml PMA. 45min after seeding, total numbers of adherent cells per mm^2^ were quantified. Analyzed were the adhesion properties of (**A**, n = 3) resting CD4^+^ T cells to fibronectin, of activated effector CD4^+^ T cells (anti-CD3/anti-CD28 antibodies for 72h) to (**C**, n = 3) ICAM-1-Fc and (**D**, n = 4) VCAM-1-Fc, of NK cells to (**E**, n = 4) ICAM-1-Fc and (**F**, n = 3) VCAM-1-Fc, of CD8^+^ T cells to (**G**, n = 3) ICAM-1-Fc and (**H**, n = 3) VCAM-1-Fc and of CD19^+^ B cells to (**I**, n = 4) ICAM-1-Fc and (**J**, n = 3) VCAM-1-Fc. (**B**, n = 3) Analysis of the static adhesion of human resting CD4^+^ T cells following 1h 45min pre-incubation with DMSO as a control or dynasore to inhibit dynamin2 activity. Before the cells were seeded on the ICAM-1-Fc coated surface, DMSO and dynasore were washed out. If indicated, cells were stimulated with 50ng/ml PMA. Relative adhesion efficiency was analyzed with PMA-stimulated control cells set to one. Mean +SEM, *P≤0.05, **P≤0.01, ***P≤0.001, ns means not significant.
(PDF)

S3 Fig Dynamin2 regulates integrin-dependent migration on 2D-VCAM-1-Fc. (**A**) Migration tracks of primary human resting CD4^+^ T lymphocytes migrating on a 2D surface coated with VCAM-1-Fc for 30min. If indicated, migration was stimulated by adding 1μg/ml CXCL12 uniformly. Cells were incubated with either DMSO as a control or dynasore to inhibit dynamin2 activity. (**B**) Quantification of accumulated distance and (**C**) average migratory speed (velocity) of the migrating lymphocytes corresponding to (**A**). Results show one representative experiment out of three. ***P≤0.001.
(PDF)

S4 Fig Dynamin2 specifically regulates Rap1 activation in human resting CD4^+^ T cells and also is essential for sustaining permanent Rap1 activity and adhesion-dependent motility in effector T cells. (**A**) Phosphorylation states of Erk1/2 and Akt were analyzed in human resting CD4^+^ T cells. Either DMSO or dynasore was added to the cells. If indicated, the lymphocytes were stimulated with 50ng/ml PMA for 15min and/or were plated on VCAM-1-Fc/ICAM-1-Fc coated surfaces. (**B**-**C**) Biochemical pull-downs of Rap1-GTP via immobilized GST-Ral-GDS-RBD were analyzed using western blotting. If indicated, cells were treated with either DMSO as a control or dynasore to inhibit dynamin2 activity. Cell lysates were generated from (**B**) primary resting human CD4^+^ T cells, which were stimulated with anti-CD3/CD28 coated beads (1:1 ratio to cells) for 2min if indicated, and from (**C**) activated CD4^+^ effector T cells (72h stimulated with anti-CD3/CD28) which were in no contact to stimulating antibodies for several hours before the experiment was carried out. (**D**-**I**) Analysis of the unstimulated motility of CD4^+^ effector T cells on a 2D surface coated with either (**D**-**F**) ICAM-1-Fc or (**G**-**I**) VCAM-1-Fc. The lymphocytes were tracked over 30min. Migration tracks as well as calculated accumulated distances and average velocities are depicted. ***P≤0.001.
(PDF)

S5 Fig Integrin surface expression and PMA-induced affinity regulation of beta_1_-integrins are not altered by dynasore in human CD4^+^ T cells. (**A**) FACS analysis of the surface expression of the beta_2_-integrin chain (CD18, n = 4) and the beta_1_-integrin chain (CD29, n = 3) on human resting CD4^+^ T cells following a 2h incubation with DMSO as a control or dynasore to inhibit dynamin2 activity. Relative expression is shown in % of mean fluorescence intensity (MFI) with DMSO control set to 100%. (**B**) FACS analysis of the surface expression of different alpha- and beta-integrin chains on effector T cells following a 2h incubation with DMSO or dynasore (histograms depict MFI). (**C**, n = 3) FACS analysis of the expression of a beta_1_-integrin activation epitope on primary human resting CD4^+^ T cells recognized by the monoclonal antibody HUTS-4. Lymphocytes were either incubated with DMSO as a control or with dynasore to inhibit dynamin2 activity. If indicated, cells were stimulated with 50ng/ml PMA for 20min. Mean fluorescence intensity (MFI) of DMSO-treated PMA-stimulated cells was set to one. Mean +SEM, *P≤0.05, **P≤0.01, ns means not significant.
(PDF)

S6 Fig Dynamin2 regulates clustering of KIM185-bound beta_2_-integrins. (**A**) Analysis of the spatial distribution of KIM185-bound (high affinity) beta_2_-integrins on polarized and unpolarized human resting CD4^+^ T cells, respectively. Representative maximum intensity projections of Z-stacks with 0.3–0.5μm interval are depicted. Z-stacks were acquired using a confocal laser scanning microscope. The cells were treated with the membrane marker Fast DIO and fluorescently labeled KIM185 antibody. (**B**) Clustering of KIM185-bound (high affinity) beta_2_-integrins on human resting CD4^+^ T cells placed on an ICAM-1-Fc coated surface was analyzed. Lymphocytes were either treated with DMSO or with dynasore. Z-stacks with 1μm interval were acquired using a confocal laser scanning microscope. Representative maximum intensity projections from different time points following seeding of the cells are depicted.
(PDF)

S1 Video Dynamin2 eGFP localizes to the basal plasma membrane in adherent Jurkat E6.1 T cells. Overexpression of human dynamin2 eGFP and lifeact RFP in a Jurkat E6.1 T cell plated in HBSS on a surface (μ-slide I) coated with ICAM-1-Fc as well as anti-CD3 and anti-CD28 antibodies. Focus on basal plasma membrane shortly after cell was exposed to the surface. Images were acquired every 5s for 5min in total using a Zeiss LSM 5 Live confocal laser scanning microscope equipped with a 100x Plan-Fluar oil immersion objective (NA 1.45) and a climate chamber (37°C). Image acquisition software was Zeiss 5 Live AIM 4.0SP2. Video was generated using ImageJ.
(MP4)

S2 Video Dynamin2 regulates integrin-mediated T lymphocyte adhesion under laminar flow. Human primary resting CD4^+^ T cells were treated with either DMSO as a control or dynasore to inhibit dynamin2 activity. Capability of the cells to adhere to a surface covered with ICAM-1-Fc/VCAM-1-Fc/CXCL12 under unidirectional laminar flow was analyzed using a custom-built flow chamber and a shear stress of 2.8 dyn/cm^2^. Phase contrast images were acquired every 10s for 30min in total using a Nikon Eclipse TE2000 microscope equipped with 20x Planfluor objective (NA 0.45) and a climate chamber (37°C). Image acquisition software was Nikon NIS Elements 2.3. Video was generated using ImageJ.
(MP4)

S3 Video Dynamin2 is essential for the T lymphocyte migration on a two-dimensional surface coated with ICAM-1-Fc. Human primary resting CD4^+^ T cells were treated with either DMSO as a control or dynasore to inhibit dynamin2 activity. Cells were seeded in HBSS on ICAM-1-Fc coated μ-slides and, if indicated, stimulated uniformly with 1μg/ml CXCL12. Phase contrast images were acquired every 15s for 30min in total using a Nikon Eclipse TE2000 microscope equipped with 10x Planfluor objective (NA 0.3), a climate chamber (37°C) and a motorized xyz-stage (Märzhäuser). Image acquisition software was Nikon NIS Elements 2.3. Video was generated using ImageJ.
(MP4)

S4 Video Dynamin2 is essential for the T lymphocyte migration on a two-dimensional surface coated with VCAM-1-Fc. Human primary resting CD4^+^ T cells were treated with either DMSO as a control or dynasore to inhibit dynamin2 activity. Cells were seeded in HBSS on VCAM-1-Fc coated μ-slides and, if indicated, stimulated uniformly with 1μg/ml CXCL12. Phase contrast images were acquired every 15s for 30min in total using a Nikon Eclipse TE2000 microscope equipped with 10x Planfluor objective (NA 0.3), a climate chamber (37°C) and a motorized xyz-stage (Märzhäuser). Image acquisition software was Nikon NIS Elements 2.3. Video was generated using ImageJ.
(MP4)

S5 Video Dynamin2 only partially regulates the migration of T cells in a complex three-dimensional collagen gel. Human primary resting CD4^+^ T cells were treated with either DMSO as a control or dynasore to inhibit dynamin2 activity. Cells were seeded a three-dimensional collagen gel and, if indicated, stimulated uniformly with 1μg/ml CXCL12. Phase contrast images were acquired every 20s for 30min in total using a Nikon Eclipse TE2000 microscope equipped with 20x Planfluor objective (NA 0.45), a climate chamber (37°C) and a motorized xyz-stage (Märzhäuser). Image acquisition software was Nikon NIS Elements 2.3. Video was generated using ImageJ.
(MP4)

S6 Video Dynamin2 regulates clustering of KIM185-bound beta_2_-integrins on T cells seeded on a surface coated with ICAM-1-Fc. Human primary resting CD4^+^ T cells were treated with either DMSO as a control or dynasore to inhibit dynamin2 activity. In addition, lymphocytes were incubated with fluorescently labeled KIM185 antibody. T cells were resuspended in HBSS and seeded in μ-slides coated with ICAM-1-Fc. After they settled, Z-stacks with an interval of 1μm were acquired every minute over 30min using a Zeiss LSM 5 Live confocal laser scanning microscope equipped with a 100x Plan-Fluar oil immersion objective (NA 1.45), a climate chamber (37°C) and a motorized xyz-stage. The video shows maximum intensity projections of the whole Z-stacks. In addition to the images showing the raw fluorescence signal of fluorescently labeled KIM185, images processed with a rainbow intensity filter are depicted. Image acquisition software was Zeiss 5 Live AIM 4.0SP2. Video was generated using ImageJ.
(MP4)

S1 Dataset Individual data points behind means, medians and variance measures presented in the figures. (XLSX)
