# Supplementary material for: Dynamin2 controls Rap1 activation and integrin clustering in human T lymphocyte adhesion
Source: PLoS One. 2017 Mar 8;12(3):e0172443. doi: 10.1371/journal.pone.0172443 (PMC5342215; doi:10.1371/journal.pone.0172443)
Supplement: S1 Fig — (A) Scheme of the experimental setup to study integrin-mediated cell adhesion under static conditions. The center of a petri dish is coated with an integrin ligand whereas the periphery is not. Phase contrast images depict boundary areas between the coated and non-coated surfaces in the petri dish with PMA-stimulated, adherent primary human resting CD4+ T cells, which strongly depend on the presence of an integrin ligand to be able to adhere firmly to the surface. (B) Phase contrast images of human resting CD4+ T cells adherent to ICAM-1-Fc. The cells need to be stimulated (in this case with 50ng/ml PMA) in order to adhere to the provided integrin ligand. (C) Semi-quantitative PCR analysis of the mRNA expression of dynamin1, dynamin2 and dynamin3 in primary resting human CD4+ T cells. Mean +SEM, *** P≤0.001, n = 5. (D) Confocal images depicting a Jurkat E6.1 T cell overexpressing human dynamin2 eGFP and lifeact RFP. The cell is plated on a surface coated with ICAM-1-Fc as well as anti-CD3 (10μg/ml) and anti-CD28 (20μg/ml) antibodies. Focus on basal plasma membrane. (PDF) [file pone.0172443.s001.pdf]

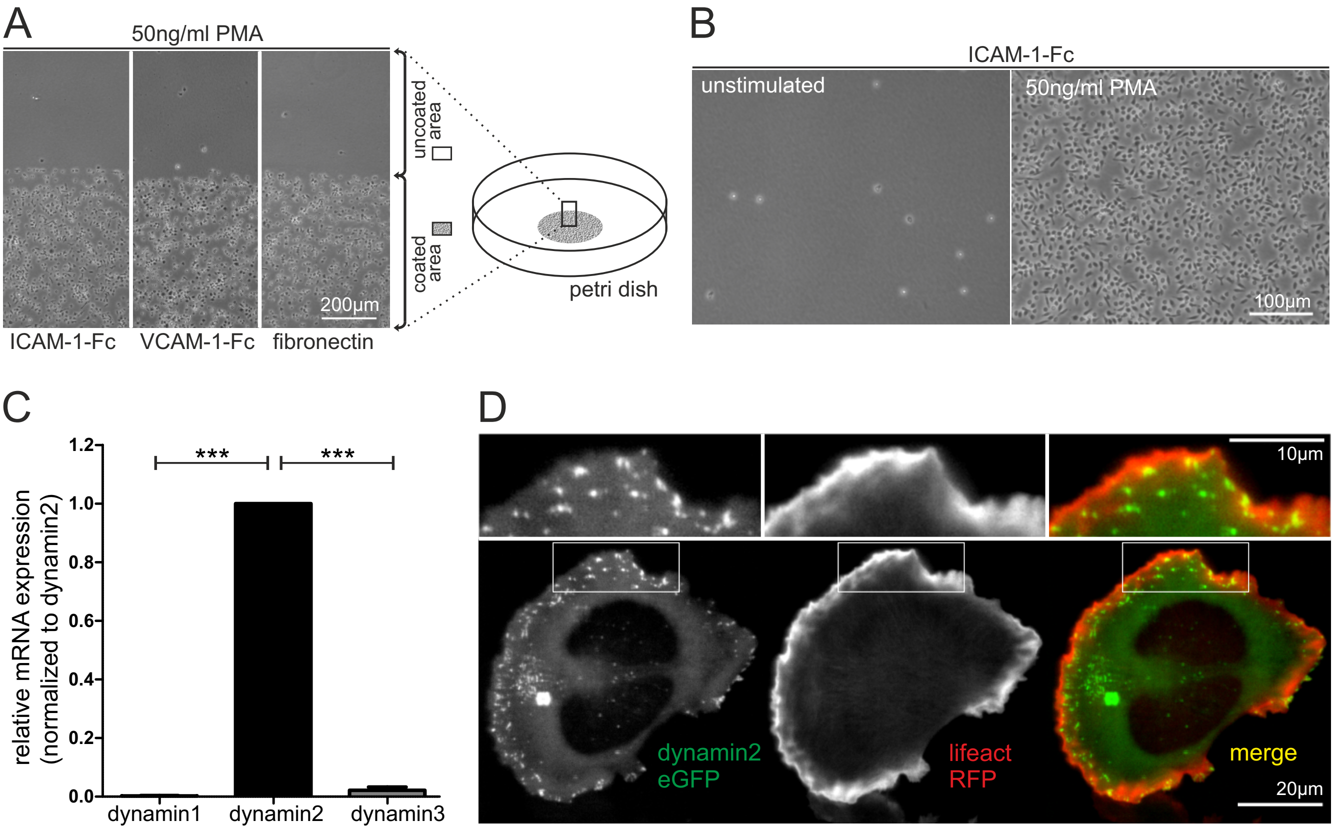

S1 Figure. Human T cells are a good model system to study integrin-mediated adhesion and they strongly express dynamin2. (**A**) Scheme of the experimental setup to study integrin-mediated cell adhesion under static conditions. The center of a petri dish is coated with an integrin ligand whereas the periphery is not. Phase contrast images depict boundary areas between the coated and non-coated surfaces in the petri dish with PMA-stimulated, adherent primary human resting $CD4^+$ T cells, which strongly depend on the presence of an integrin ligand to be able to adhere firmly to the surface. (**B**) Phase contrast images of human resting $CD4^+$ T cells adherent to ICAM-1-Fc. The cells need to be stimulated (in this case with 50ng/ml PMA) in order to adhere to the provided integrin ligand. (**C**) Semi-quantitative PCR analysis of the mRNA expression of dynamin1, dynamin2 and dynamin3 in primary resting human $CD4^+$ T cells. Mean +SEM, *** P≤0.001, n=5. (**D**) Confocal images depicting a Jurkat E6.1 T cell overexpressing human dynamin2 eGFP and lifeact RFP. The cell is plated on a surface coated with ICAM-1-Fc as well as anti-CD3 (10µg/ml) and anti-CD28 (20µg/ml) antibodies. Focus on basal plasma membrane.
