# Supplementary material for: Dynamin2 controls Rap1 activation and integrin clustering in human T lymphocyte adhesion
Source: PLoS One. 2017 Mar 8;12(3):e0172443. doi: 10.1371/journal.pone.0172443 (PMC5342215; doi:10.1371/journal.pone.0172443)
Supplement: S2 Fig — (A, C-J) Analysis of the adhesion of different types of primary human lymphocytes to either fibronectin, ICAM-1-Fc or VCAM-1-Fc under static conditions. Lymphocytes were treated with DMSO as a control or 80μM dynasore to inhibit dynamin2 activity. If indicated, adhesion was stimulated with 50ng/ml PMA. 45min after seeding, total numbers of adherent cells per mm2 were quantified. Analyzed were the adhesion properties of (A, n = 3) resting CD4+ T cells to fibronectin, of activated effector CD4+ T cells (anti-CD3/anti-CD28 antibodies for 72h) to (C, n = 3) ICAM-1-Fc and (D, n = 4) VCAM-1-Fc, of NK cells to (E, n = 4) ICAM-1-Fc and (F, n = 3) VCAM-1-Fc, of CD8+ T cells to (G, n = 3) ICAM-1-Fc and (H, n = 3) VCAM-1-Fc and of CD19+ B cells to (I, n = 4) ICAM-1-Fc and (J, n = 3) VCAM-1-Fc. (B, n = 3) Analysis of the static adhesion of human resting CD4+ T cells following 1h 45min pre-incubation with DMSO as a control or dynasore to inhibit dynamin2 activity. Before the cells were seeded on the ICAM-1-Fc coated surface, DMSO and dynasore were washed out. If indicated, cells were stimulated with 50ng/ml PMA. Relative adhesion efficiency was analyzed with PMA-stimulated control cells set to one. Mean +SEM, *P≤0.05, **P≤0.01, ***P≤0.001, ns means not significant. (PDF) [file pone.0172443.s002.pdf]

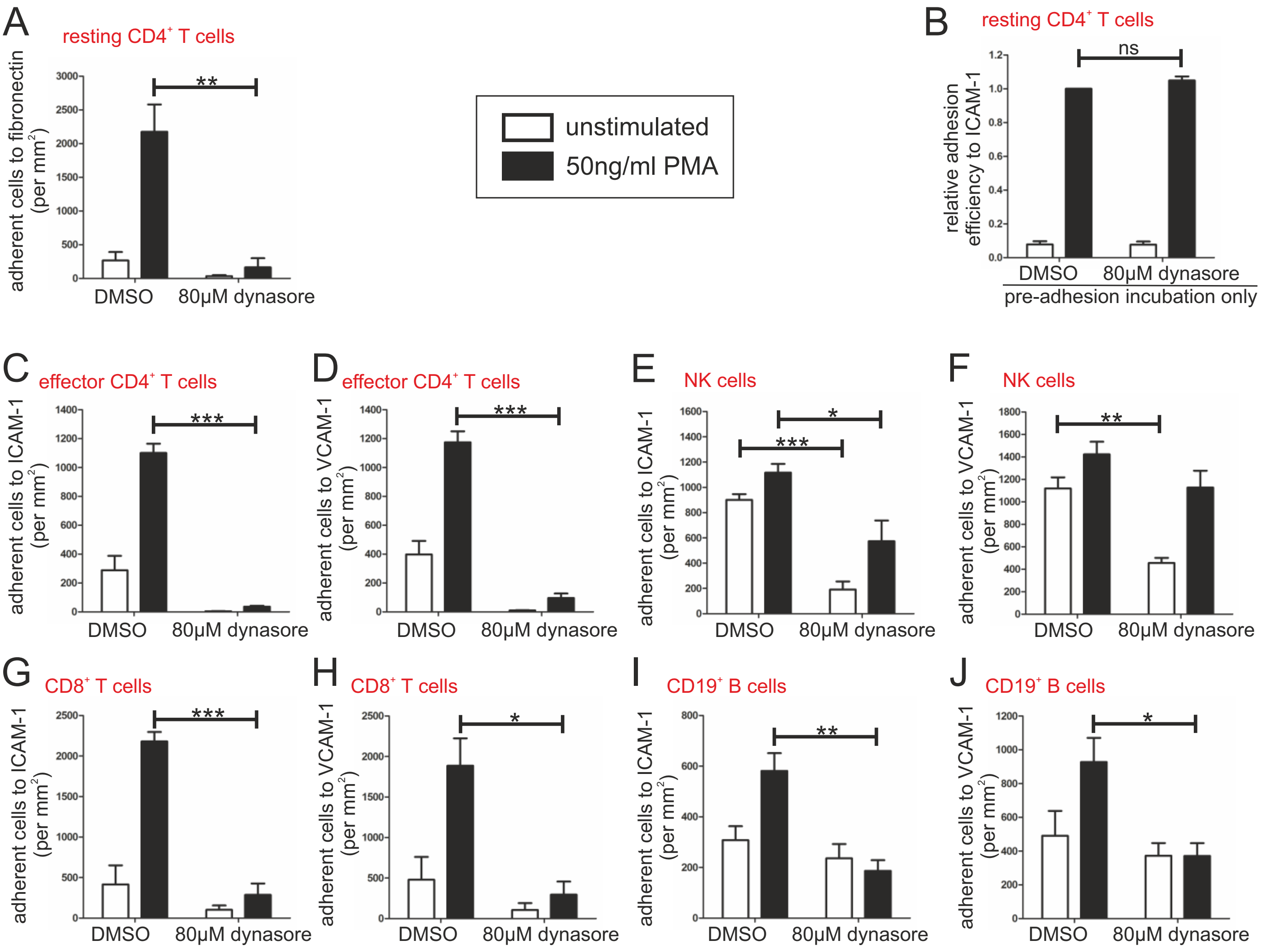

S2 Figure. Integrin-mediated adhesion of human lymphocytes strongly depends on dynamin2 activity. (**A, C-J**) Analysis of the adhesion of different types of primary human lymphocytes to either fibronectin, ICAM-1-Fc or VCAM-1-Fc under static conditions. Lymphocytes were treated with DMSO as a control or 80µM dynasore to inhibit dynamin2 activity. If indicated, adhesion was stimulated with 50ng/ml PMA. 45min after seeding, total numbers of adherent cells per mm^2 were quantified. Analyzed were the adhesion properties of (**A**, n=3) resting $CD4^+$ T cells to fibronectin, of activated effector $CD4^+$ T cells (anti-CD3/anti-CD28 antibodies for 72h) to (**C**, n=3) ICAM-1-Fc and (**D**, n=4) VCAM-1-Fc, of NK cells to (**E**, n=4) ICAM-1-Fc and (**F**, n=3) VCAM-1-Fc, of $CD8^+$ T cells to (**G**, n=3) ICAM-1-Fc and (**H**, n=3) VCAM-1-Fc and of $CD19^+$ B cells to (**I**, n=4) ICAM-1-Fc and (**J**, n=3) VCAM-1-Fc. (**B**, n=3) Analysis of the static adhesion of human resting $CD4^+$ T cells following 1h 45min pre-incubation with DMSO as a control or dynasore to inhibit dynamin2 activity. Before the cells were seeded on the ICAM-1-Fc coated surface, DMSO and dynasore were washed out. If indicated, cells were stimulated with 50ng/ml PMA. Relative adhesion efficiency was analyzed with PMA-stimulated control cells set to one. Mean +SEM, *P≤0.05, **P≤0.01, ***P≤0.001, ns means not significant.
