# Supplementary material for: Dynamin2 controls Rap1 activation and integrin clustering in human T lymphocyte adhesion
Source: PLoS One. 2017 Mar 8;12(3):e0172443. doi: 10.1371/journal.pone.0172443 (PMC5342215; doi:10.1371/journal.pone.0172443)
Supplement: S3 Fig — (A) Migration tracks of primary human resting CD4+ T lymphocytes migrating on a 2D surface coated with VCAM-1-Fc for 30min. If indicated, migration was stimulated by adding 1μg/ml CXCL12 uniformly. Cells were incubated with either DMSO as a control or dynasore to inhibit dynamin2 activity. (B) Quantification of accumulated distance and (C) average migratory speed (velocity) of the migrating lymphocytes corresponding to (A). Results show one representative experiment out of three. ***P≤0.001. (PDF) [file pone.0172443.s003.pdf]

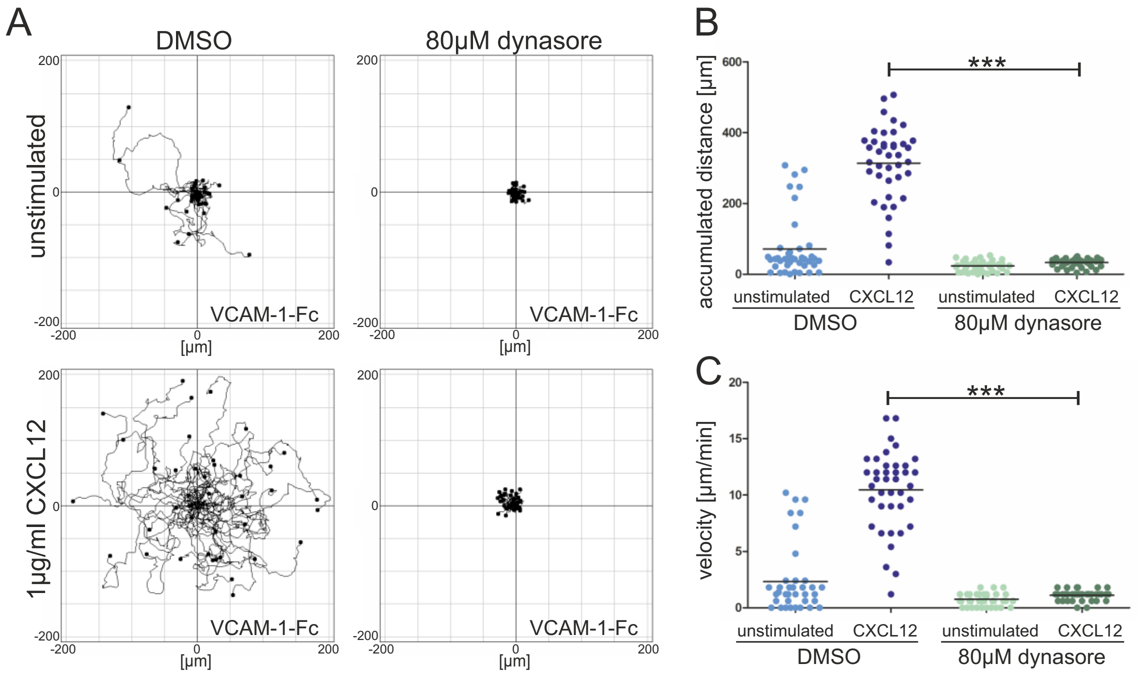

S3 Figure. Dynamin2 regulates integrin-dependent migration on 2D-VCAM-1-Fc. (**A**) Migration tracks of primary human resting CD4[+] T lymphocytes migrating on a 2D surface coated with VCAM-1-Fc for 30min. If indicated, migration was stimulated by adding 1µg/ml CXCL12 uniformly. Cells were incubated with either DMSO as a control or dynasore to inhibit dynamin2 activity. (**B**) Quantification of accumulated distance and (**C**) average migratory speed (velocity) of the migrating lymphocytes corresponding to (A). Results show one representative experiment out of three. ***P≤0.001.
