# Supplementary material for: Dynamin2 controls Rap1 activation and integrin clustering in human T lymphocyte adhesion
Source: PLoS One. 2017 Mar 8;12(3):e0172443. doi: 10.1371/journal.pone.0172443 (PMC5342215; doi:10.1371/journal.pone.0172443)
Supplement: S4 Fig — (A) Phosphorylation states of Erk1/2 and Akt were analyzed in human resting CD4+ T cells. Either DMSO or dynasore was added to the cells. If indicated, the lymphocytes were stimulated with 50ng/ml PMA for 15min and/or were plated on VCAM-1-Fc/ICAM-1-Fc coated surfaces. (B-C) Biochemical pull-downs of Rap1-GTP via immobilized GST-Ral-GDS-RBD were analyzed using western blotting. If indicated, cells were treated with either DMSO as a control or dynasore to inhibit dynamin2 activity. Cell lysates were generated from (B) primary resting human CD4+ T cells, which were stimulated with anti-CD3/CD28 coated beads (1:1 ratio to cells) for 2min if indicated, and from (C) activated CD4+ effector T cells (72h stimulated with anti-CD3/CD28) which were in no contact to stimulating antibodies for several hours before the experiment was carried out. (D-I) Analysis of the unstimulated motility of CD4+ effector T cells on a 2D surface coated with either (D-F) ICAM-1-Fc or (G-I) VCAM-1-Fc. The lymphocytes were tracked over 30min. Migration tracks as well as calculated accumulated distances and average velocities are depicted. ***P≤0.001. (PDF) [file pone.0172443.s004.pdf]

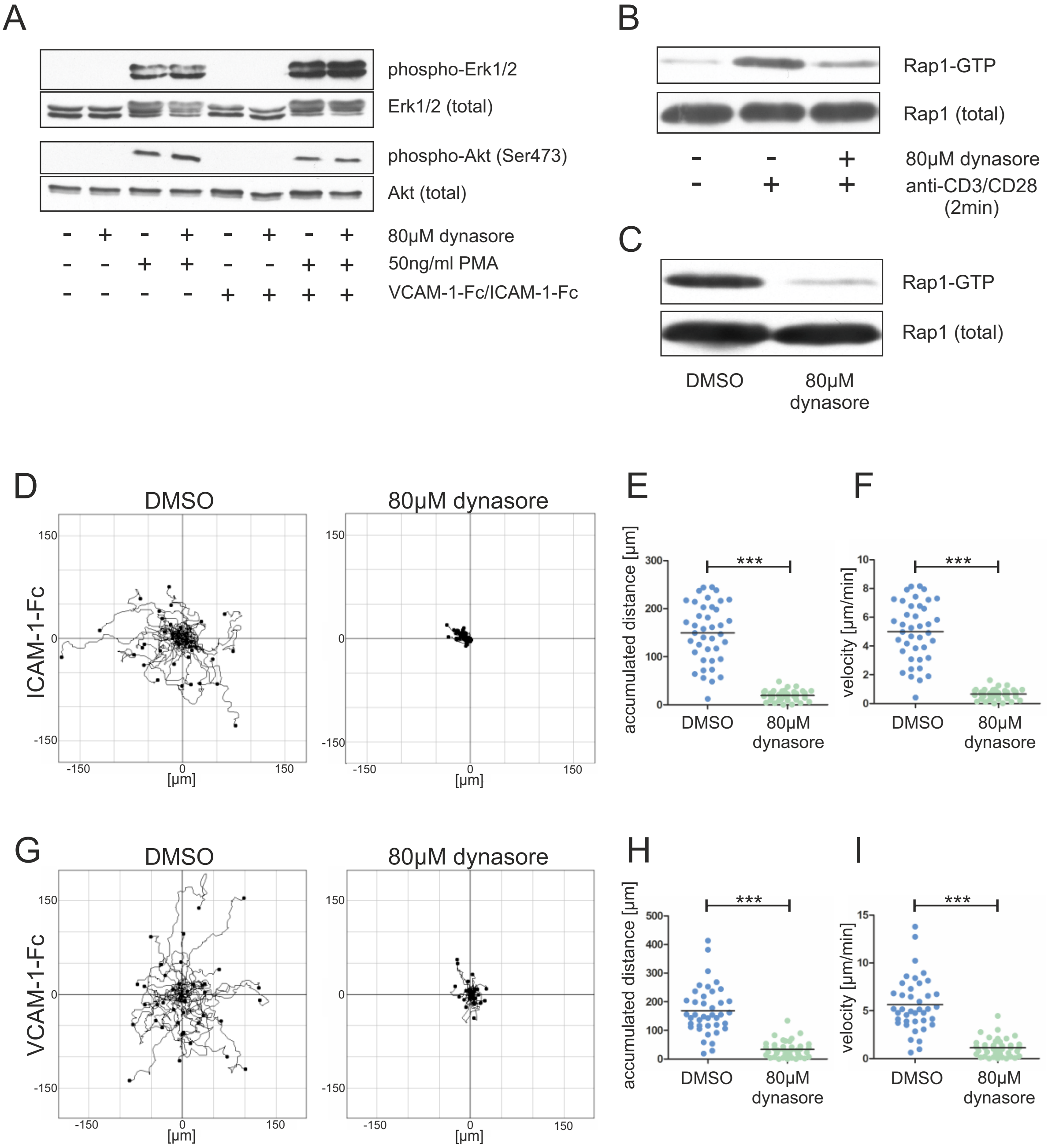

S4 Figure. Dynamin2 specifically regulates Rap1 activation in human resting CD4⁺ T cells and also is essential for sustaining permanent Rap1 activity and adhesion-dependent motility in effector T cells. (**A**) Phosphorylation states of Erk1/2 and Akt were analyzed in human resting CD4⁺ T cells. Either DMSO or dynasore was added to the cells. If indicated, the lymphocytes were stimulated with 50ng/ml PMA for 15min and/or were plated on VCAM-1-Fc/ICAM-1-Fc coated surfaces. (**B-C**) Biochemical pull-downs of Rap1-GTP via immobilized GST-Ral-GDS-RBD were analyzed using western blotting. If indicated, cells were treated with either DMSO as a control or dynasore to inhibit dynamin2 activity. Cell lysates were generated from (**B**) primary resting human CD4⁺ T cells, which were stimulated with anti-CD3/CD28 coated beads (1:1 ratio to cells) for 2min if indicated, and from (**C**) activated CD4⁺ effector T cells (72h stimulated with anti-CD3/CD28) which were in no contact to stimulating antibodies for several hours before the experiment was carried out. (**D-I**) Analysis of the unstimulated motility of CD4⁺ effector T cells on a 2D surface coated with either (**D-F**) ICAM-1-Fc or (**G-I**) VCAM-1-Fc. The lymphocytes were tracked over 30min. Migration tracks as well as calculated accumulated distances and average velocities are depicted. ***P≤0.001.
