# Supplementary material for: Dynamin2 controls Rap1 activation and integrin clustering in human T lymphocyte adhesion
Source: PLoS One. 2017 Mar 8;12(3):e0172443. doi: 10.1371/journal.pone.0172443 (PMC5342215; doi:10.1371/journal.pone.0172443)
Supplement: S5 Fig — (A) FACS analysis of the surface expression of the beta2-integrin chain (CD18, n = 4) and the beta1-integrin chain (CD29, n = 3) on human resting CD4+ T cells following a 2h incubation with DMSO as a control or dynasore to inhibit dynamin2 activity. Relative expression is shown in % of mean fluorescence intensity (MFI) with DMSO control set to 100%. (B) FACS analysis of the surface expression of different alpha- and beta-integrin chains on effector T cells following a 2h incubation with DMSO or dynasore (histograms depict MFI). (C, n = 3) FACS analysis of the expression of a beta1-integrin activation epitope on primary human resting CD4+ T cells recognized by the monoclonal antibody HUTS-4. Lymphocytes were either incubated with DMSO as a control or with dynasore to inhibit dynamin2 activity. If indicated, cells were stimulated with 50ng/ml PMA for 20min. Mean fluorescence intensity (MFI) of DMSO-treated PMA-stimulated cells was set to one. Mean +SEM, *P≤0.05, **P≤0.01, ns means not significant. (PDF) [file pone.0172443.s005.pdf]

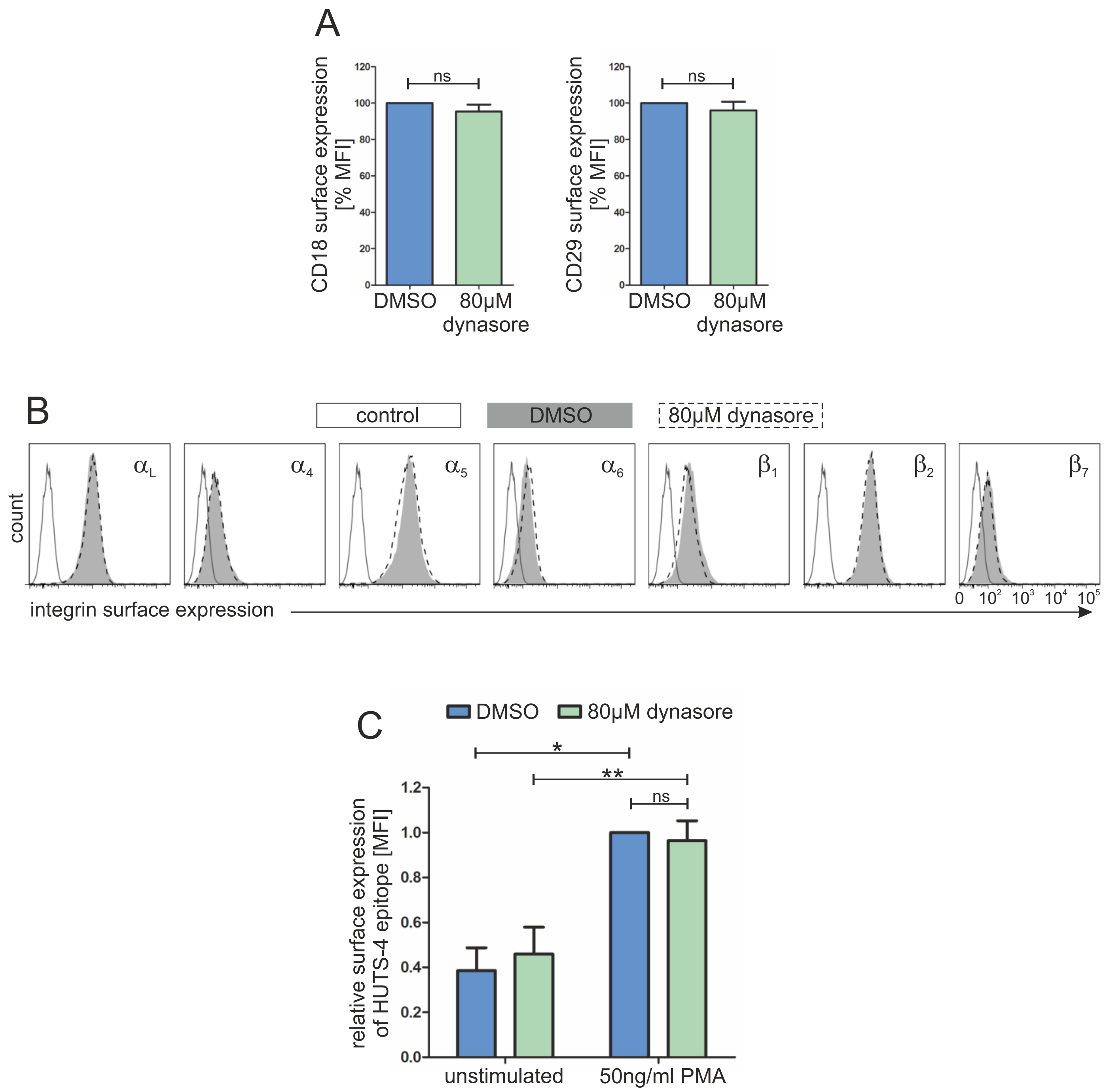


**S5 Figure. Integrin surface expression and PMA-induced affinity regulation of beta$_1$-integrins are not altered by dynasore in human CD4$^+$ T cells.** (**A**) FACS analysis of the surface expression of the beta$_2$-integrin chain (CD18, n=4) and the beta$_1$-integrin chain (CD29, n=3) on human resting CD4$^+$ T cells following a 2h incubation with DMSO as a control or dynasore to inhibit dynamin2 activity. Relative expression is shown in % of mean fluorescence intensity (MFI) with DMSO control set to 100%. (**B**) FACS analysis of the surface expression of different alpha- and beta-integrin chains on effector T cells following a 2h incubation with DMSO or dynasore (histograms depict MFI). (**C**, n=3) FACS analysis of the expression of a beta$_1$-integrin activation epitope on primary human resting CD4$^+$ T cells recognized by the monoclonal antibody HUTS-4. Lymphocytes were either incubated with DMSO as a control or with dynasore to inhibit dynamin2 activity. If indicated, cells were stimulated with 50ng/ml PMA for 20min. Mean fluorescence intensity (MFI) of DMSO-treated PMA-stimulated cells was set to one. Mean +SEM, *P≤0.05, **P≤0.01, ns means not significant.
