# Supplementary material for: Dynamin2 controls Rap1 activation and integrin clustering in human T lymphocyte adhesion
Source: PLoS One. 2017 Mar 8;12(3):e0172443. doi: 10.1371/journal.pone.0172443 (PMC5342215; doi:10.1371/journal.pone.0172443)
Supplement: S6 Fig — (A) Analysis of the spatial distribution of KIM185-bound (high affinity) beta2-integrins on polarized and unpolarized human resting CD4+ T cells, respectively. Representative maximum intensity projections of Z-stacks with 0.3–0.5μm interval are depicted. Z-stacks were acquired using a confocal laser scanning microscope. The cells were treated with the membrane marker Fast DIO and fluorescently labeled KIM185 antibody. (B) Clustering of KIM185-bound (high affinity) beta2-integrins on human resting CD4+ T cells placed on an ICAM-1-Fc coated surface was analyzed. Lymphocytes were either treated with DMSO or with dynasore. Z-stacks with 1μm interval were acquired using a confocal laser scanning microscope. Representative maximum intensity projections from different time points following seeding of the cells are depicted. (PDF) [file pone.0172443.s006.pdf]

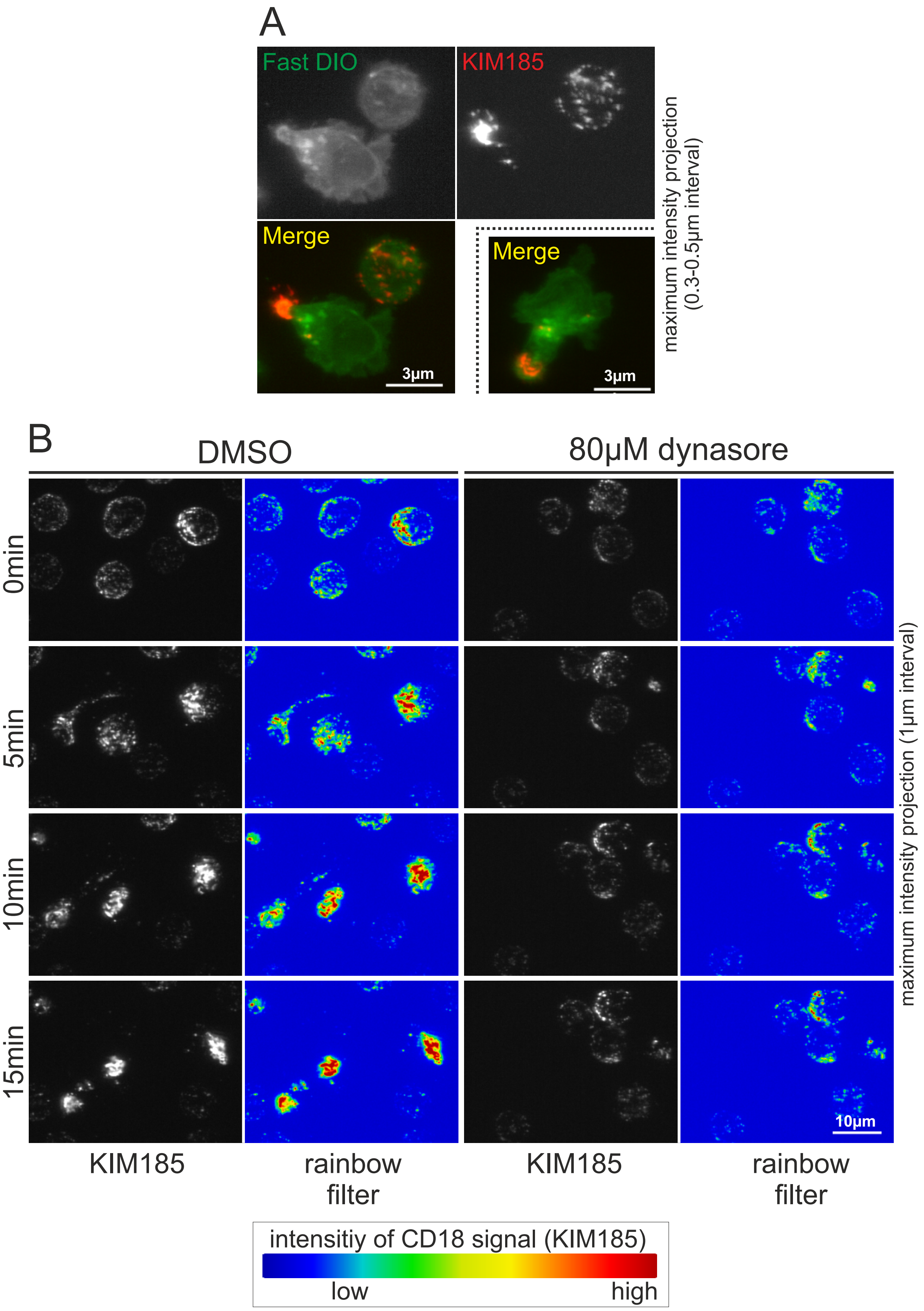

S6 Figure. Dynamin2 regulates clustering of KIM185-bound beta$_2$-integrins. (**A**) Analysis of the spatial distribution of KIM185-bound (high affinity) beta$_2$-integrins on polarized and unpolarized human resting CD4$^+$ T cells, respectively. Representative maximum intensity projections of Z-stacks with 0.3-0.5µm interval are depicted. Z-stacks were acquired using a confocal laser scanning microscope. The cells were treated with the membrane marker Fast DIO and fluorescently labeled KIM185 antibody. (**B**) Clustering of KIM185-bound (high affinity) beta$_2$-integrins on human resting CD4$^+$ T cells placed on an ICAM-1-Fc coated surface was analyzed. Lymphocytes were either treated with DMSO or with dynasore. Z-stacks with 1µm interval were acquired using a confocal laser scanning microscope. Representative maximum intensity projections from different time points following seeding of the cells are depicted.
